# Update of guidelines on laparoscopic (TAPP) and endoscopic (TEP) treatment of inguinal hernia (International Endohernia Society)

**DOI:** 10.1007/s00464-014-3917-8

**Published:** 2014-11-15

**Authors:** R. Bittner, M. A. Montgomery, E. Arregui, V. Bansal, J. Bingener, T. Bisgaard, H. Buhck, M. Dudai, G. S. Ferzli, R. J. Fitzgibbons, R. H. Fortelny, K. L. Grimes, U. Klinge, F. Koeckerling, S. Kumar, J. Kukleta, D. Lomanto, M. C. Misra, S. Morales-Conde, W. Reinpold, J. Rosenberg, K. Singh, M. Timoney, D. Weyhe, P. Chowbey

**Affiliations:** 1 Hernia Center, Winghofer Medicum Plus, Röntgenstr. 38, 72108 Rottenburg, Germany; 2 Section of Laparoscopy and Abdominal Wall Reconstruction, Department of Surgery, Skåne University Hospital, 20502 Malmö, Sweden; 3 Advanced GI Surgery, Laparoscopy, Endoscopy and Ultrasound, St. Vincent Hospital and Health Care Center, 8402 Harcourt Rd. Suite 815, Indianapolis, IN 46260 USA; 4 Division of Minimally Invasive Surgery, Department of Surgical Disciplines, All India Institute of Medical Sciences, Angari Nagar, New Delhi, 110029 India; 5 Division of Gastroenterological and General Surgery, Mayo Clinic, 200 First Street SW, Rochester, MN 55905 USA; 6 Gastro Unit, Surgical Section, Hvidovre Hospital, University of Copenhagen, Copenhagen, Denmark; 7 Med Comm Tools, Hildesheimer Str. 13, 30169 Hannover, Germany; 8 Department of Surgery, Ramat Aviv Medical Center, Tel Aviv, Israel; 9 Department of Surgery, Lutheran Medical Center, SUNY Health Science Center, Brooklyn, 65 Cromwell Avenue, Staten Island, NY USA; 10 Department of Surgery, Creighton University, 601 North 30th Street, Suite 3700, Omaha, NE USA; 11 Department of General, Visceral and Oncological Surgery, Wilhelminenspital, 1171 Vienna, Austria; 12 Surgical Department, University of Aachen, and Institute for Applied Medical Engineering AME Helmholtz, Pauwelstrasse, 52074 Aachen Germany; 13 Department of Surgery and Center for Minimally Invasive Surgery, Vivantes Hospital, Neue Bergstr. 6, 13585 Berlin, Germany; 14 General, Visceral, Abdominal Wall Surgery, Klinik Im Park, Grossmuensterplatz 9, 8001 Zurich, Switzerland; 15 Minimally Invasive Surgical Center, KTP Advanced Surgical Training Center, YYL School of Medicine, National University Hospital, Kent Ridge Wing 2, 5 Lower Kent Ridge Road, Singapore, 119074 Singapore; 16 Advanced Laparoscopic Unit of the University Hospital “Virgen del Rocío”, General, Digestive and Laparoscopic Surgery Unit of the USP-“Sagrado Corazón” Clinic, University of Sevilla, Seville, Spain; 17 Department of Surgery, Gross-Sand Hospital Hamburg, Gross-Sand 3, 21107 Hamburg, Germany; 18 Department of Surgery D, Herlev Hospital, University of Copenhagen, Copenhagen, Denmark; 19 Department of Surgery, Pius Hospital, Georgstrasse 12, 26121 Oldenburg, Germany; 20 Minimal Access, Metabolic, and Bariatric Surgery, Max Healthcare Institute Ltd., 2 Press Enclave Road, Saket, New Delhi, India

## Introduction

Guidelines are the bridge between science and clinical practice [1]. Science is a dynamic process and it is continuously evolving. Consequently, there is a continual development of new insights necessitation updates of existing guidelines. For this update, the authors concentrated on studies with level of 1 and 2 evidence. All references are marked with the level of evidence, according to the Oxford classification. In general “Recommendation Grade D” does not constitute a recommendation, but in some instances it is shown in the text to indicate lack of quality data. We recommended all readers to download the original statements and recommendations [2], for fully appreciation of the Update Guidelines on Laparoscopic Hernia Surgery.

Updates should include issues that were not yet sufficiently covered in the original guidelines or those which have gained increased clinical importance. For this reason, the Update includes four new chapters: single port surgery, convalescence, costs and training. The update process was started in March 2013. All the authors were requested to commence revision of their chapters between January 2009 and September 30th 2013. An Update Consensus Conference was held on October 23–26, 2013 in Windhoek/Namibia, following which, the first versions of the updates were presented to the delegates and extensively discussed. Based on these discussions the definite update was formulated and circulated for approval by all the involved experts.

## References (in parentheses graduation of evidence)

1. Eccles M, Mason J (2001) How to develop cost-conscious guidelines. Health Technol Assess 5(16):1–69. Reviews
2. Bittner R, Arregui ME, Bisgaard T, Dudai M, Ferzli GS, Fitzgibbons RJ, Fortelny RH, Klinge U, Kockerling F, Kuhry E, Kukleta J, Lomanto D, Misra MC, Montgomery A, Morales-Conde S, Reinpold W, Rosenberg J, Sauerland S, Schug-Pass C, Singh K, Timoney M, Weyhe D, Chowbey P (2011) Guidelines for laparoscopic (TAPP) and endoscopic (TEP) treatment of inguinal hernia [International Endohernia Society (IEHS)]. Surg Endosc 25(9):2773–284

## Chapter 1: Perioperative management: evidence for antibiotic and thromboembolic prophylaxis in endoscopic/laparoscopic inguinal hernia surgery?

### Agneta montgomery

*Antibiotic prophylaxis*

**Search terms**: “Antibiotic prophylaxis*” AND “laparoscopy” AND “inguinal hernia”; “Antibiotic prophylaxis*” AND “TEP”; “Antibiotic prophylaxis*” AND “TAPP”; “Antibiotic prophylaxis*” AND “randomized controlled trial” AND “inguinal hernia”; “Antibiotic prophylaxis*” AND “meta-analysis” AND “inguinal hernia”.

*Thromboembolic prophylaxis*

“Thromboembolic prophylaxis*” AND “laparoscopy” AND “inguinal hernia; “Thromboembolic prophylaxis*” AND “TEP”; “Thromboembolic prophylaxis*” AND “TAPP”; “Thromboembolic prophylaxis*” AND “randomized controlled trial” AND “inguinal hernia”; “Thromboembolic prophylaxis*” AND “meta-analysis” AND “inguinal hernia”.

**Search machines**

PubMed and the Cochrane Colorectal Cancer Group specialized register and reference lists of the included studies were search for studies for potential inclusion.

**New publications**

A total of 45 studies were identified as Level 1 or Level 2. No RCTs including TEP or TAPP with antibiotic or thromboembolic prophylaxis as primary outcome were identified. Three RCT studies on TEP or TAPP, having antibiotic treatment in the protocol and including more than 200 patients, were identified [1, 2, 3]. The first compared TEP to Lichtenstein [1] and the other two compared different mesh types in TAPP repair [2, 3]. Two reported on thromboembolic complications [2, 3]. Four meta-analyses on antibiotic prophylaxis for prevention of surgical site infections as a primary outcome were identified [4–7]. All included only open hernia repairs. No meta-analyses on thromboembolic complications were identified.

**Antibiotic prophylaxis**

No new statements or recommendations.

**Thromboembolic prophylaxis**

No new statements or recommendations.

**Comments**

An update of the Cochrane report analyzing open hernia repairs, non-mesh and mesh repairs, was published in 2012 (search until October 2011) including 7,843 hernia operations in 17 studies [4]. The overall infection rates were 3.1 % in the prophylaxis group and 4.5 % in the control group (OR 0.64, 95 % CI 0.50–0.82). The subgroup with mesh had infection rates of 2.4 and 4.2 % in the prophylaxis and control groups, respectively (OR 0.56, 95 % CI 0.38–0.81). The recommendation in this report was: “Antibiotic prophylaxis for elective inguinal hernia repair cannot be universally recommended for open hernia repair. Neither can the administration be recommended against when high rates of wound infection are observed.”

The three other meta-analyses are all performed on mesh repairs and all except one study is included in the Cochrane report [5–7]. They all conclude that antibiotic prophylaxis is beneficial for protection of surgical site infections in open mesh repair.

## References (in parentheses graduation of evidence)

1. Langeveld HR, van’t Riet M, Weidema WF, Stassen LP, Steyerberg EW, Lange J, Bonjer HJ, Jeekel J (2010) Total extraperitoneal inguinal hernia repair compared with Lichtenstein (the LEVEL-Trial): a randomized controlled trial. Ann Surg 251(5):819–824. (**1B**)
2. Bittner R, Schmedt CG, Leibl BJ, Schwarz J (2011) Early postoperative and one year results of a randomized controlled trial comparing the impact of extralight titanized polypropylene mesh and traditional heavyweight polypropylene mesh on pain and seroma production in laparoscopic hernia repair (TAPP). World J Surg 35(8):1791–1797. (**1B**)
3. Bittner R, Leibl BJ, Kraft B, Schwarz J (2011) One-year results of a prospective, randomised clinical trial comparing four meshes in laparoscopic inguinal hernia repair (TAPP). Hernia 15(5):503–510. (**1B**)
4. Sanchez-Manuel FJ, Lozano-García J, Seco-Gil JL (2012) Antibiotic prophylaxis for hernia repair. Cochrane Database Syst Rev 2012 Issue 2. Art. CD003769. doi: 10.1002/14651858.CD003769.pub4. (**1A**)
5. Mazaki T, Mado K, Masuda H, Shiono M (2013) Antibiotic prophylaxis for the prevention of surgical site infection after tension-free hernia repair: a Bayesian and frequentist meta-analysis. J Am Coll Surg 217(5):788–801. (**1A**)
6. Li JF, Lai DD, Zhang XD, Zhang AM, Sun KX, Luo HG, Yu Z (2012) Meta-analysis of the effectiveness of prophylactic antibiotics in the prevention of postoperative complications after tension-free hernioplasty. Can J Surg 55(1):27–32. (**1A**)
7. Yin Y, Song T, Liao B, Luo Q, Zhou Z (2012) Antibiotic prophylaxis in patients undergoing open mesh repair of inguinal hernia: a meta-analysis. Am Surg 78(3):359–365. (**1A**)

## Chapter 2: Technical key points in TAPP repair

### Jan F. Kukleta, Reinhard Bittner

**Search terms**: “Inguinal hernia“, “TAPP repair“, “TAPP”, “TAPP technique”, “hernia repair”, “endoscopic repair”. Filters: Engl., Ger., Ital., French, Port., Span. RCT, Meta-analysis, multicenter study, systematic review, controlled trial.

**Search machines**

PubMed, Medline and reference lists of articles selected for inclusion.

**New publications**

Of 1,684 papers involved with “endoscopic repair”, to “TAPP Hernia” with 355 and TAPP repair with 305. Of the 176 contributions to “TAPP technique” 37 were published in the last 3 years. (18 RCT’s, 3 meta-analysis and 16 reviews).

**Comments**

Due to the present structure of the guidelines some of the fundamental technical key points of TAPP repair like the mesh choice, mesh size, slitting/non-slitting and fixation /non fixation are discussed in depth in other chapters. These key points do influence obviously the patient’s outcome and represent an important part of the TAPP’s best practice.

In several instances Recommendation Grade D is mentioned. In general “Recommendation Grade D” is no recommendation at all, due to weak evidence. Nevertheless it is used in this text to demonstrate that some important data are still missing.

**Which is the safest and most effective method of establishing pneumoperitoneum and obtaining access to the abdominal cavity?**

New statements—identical to previous except statement below.

**Table:** 

Level 1B	In thin patients (BMI < 27), the direct trocar insertion is a safe alternative to the Veress needle technique (**stronger evidence**).

New recommendations—identical to previous except recommendation below.

**Table:** 

Grade C	The direct trocar insertion (DTI) can be used in order to establish pneumoperitoneum as a safe alternative to Veress needle, Hasson approach or optical trocar, if patient’s risk factors are considered and the surgeon is appropriately trained (**new recommendation**).

**What kind of trocars should be used?**

**Is there any relation between the trocar type and risk of injury and/or trocar hernias?**

New statement—identical to previous except statement below.

**Table:** 

Level 2B	Use of 10-mm trocars or larger may predispose to hernias, especially in the umbilical region or in the oblique abdominal wall (**Stronger evidence**).

New recommendation—identical to previous except recommendation below.

**Table:** 

Grade B	Fascial defects of 10 mm or bigger should be closed (**Stronger evidence**).

**Is clinical examination efficient enough?**

**What is the role of TAPP and other techniques in reliable assessment?**

New statements—identical to previous (but additional references, see comment).

New recommendations—identical to previous.

**Peritoneal closure**

New statements—identical to previous.

New recommendations—identical to previous except statement below.

**Table:** 

Grade B	A thorough closure of peritoneal incision or bigger peritoneal tears should be achieved (**Stronger evidence**).

**Comments**

After more than two decades of practicing TAPP repair, the technique per se is standardized to a great extent. Although only minimal changes in evidence levels and no completely new insights were to be expected in the time frame of the last 3 years, the content of the guidelines must be periodically scrutinized, re-examined and if necessary corrected. In order to reinforce the validity of existing recommendations and to improve the adoption of it by the world-wide surgical community it was sometimes necessary to interpret the evidence to make it fit better to everyday life. In paragraph “Which is the safest and most effective method of establishing pneumoperitoneum and obtaining access to the abdominal cavity?” there is a new input of stronger evidence (1B) [2] and 2B [3] concerning the direct trocar insertion. Nevertheless the authors defend their recommendation Grade A “When establishing pneumoperitoneum … extreme caution is required”. Because of the potential risk of a major injury the recommendation based on the statement “the direct trocar insertion is a safe alternative to the Veress needle technique” is intentionally downgraded to grade C: The direct trocar insertion (DTI) can be used in order to establish pneumoperitoneum as a safe alternative to Veress needle, open access or optical trocar, if patient’s risk factors are considered and the surgeon is appropriately trained.

The paragraph “What kind of trocars should be used? Is there any relation between the trocar type and risk of injury and/or trocar hernias?” [5–8] reached stronger evidence for to refrain from the use of cutting trocars in order to diminish the local trauma and prevent the development of possible trocar hernias. The recommendation Grade C “Trocar sites with fascial defects of 10 mm or larger can be closed” was upgraded to Grade B “Fascial defects of 10 mm or bigger should be closed.

The recommendations concerning clinical examination and anticipation of undiagnosed contralateral hernias gained additional support and insight from literature [9–11].

The previous recommendation on peritoneal closure already connoted verbally the importance of the task, although assigned to Grade C. To emphasize the fact the recommendation was upgraded to Grade B.

## References (in parentheses graduation of evidence)

1. Ahmad G, O’Flynn H, Duffy JM, Phillips K, Watson A (2012) Laparoscopic entry techniques. Cochrane Database Syst Rev 2:CD006583. doi: 10.1002/14651858.CD006583.pub3. (**1A**)
2. Jiang X, Anderson C, Schnatz PF (2012) The safety of direct trocar versus Veress needle for laparoscopic entry: a meta-analysis of randomized clinical trials. J Laparoendosc Adv Surg Tech 22(4):362–370. (**1B**)
3. Agresta F, Mazzarolo G, Bedin N (2012) Direct trocar insertion for laparoscopy. JSLS 16:255–259. (**2B**)
4. Dunne N, Booth MI, Dehn TCB (2011) Establishing pneumoperitoneum: Verres or Hasson? The debate continues. Ann R Coll Surg Engl 93: 22–24. (**3**)
5. Owens M, Barry M, Janjua AZ, Winter DC (2011) A systematic review of laparoscopic port site hernias in gastrointestinal surgery. Surgeon 9(4):218–224. (**2A**)
6. Helgstrand F, Rosenberg J, Kehlet H, Bisgaard T (2011) Low risk of trocar site hernia repair 12 years after primary laparoscopic surgery. Surg Endosc 25:3678–3682. doi: 10.1007/s00464-011-1776-0. (**2A**)
7. Mordecai SC, Warren OWN, Warren SJ (2012) Radially expanding laparoscopic trocar ports significantly reduce postoperative pain in all age groups. Surg Endosc 26:843–846. doi: 10.1007/s00464-011-1963-z. (**3**)
8. Bhoyrul S, Payne J, Steffes B, Swanstrom L, Way LW (2000). A randomized prospective study of radially expanding trocars in laparoscopic surgery. J Gastrointest Surg 4(4):392–397. (**2B**)
9. Griffin KJ, Harris S, Tang TY, Skelton N, Reed JB, Harris AM (2010) Incidence of contralateral occult inguinal hernia found at the time of laparoscopic trans-abdominal pre-peritoneal (TAPP) repair. Hernia 14(4):345–349. doi: 10.1007/s10029-010-0651-6. (**2B**)
10. van den Heuvel B, Beudeker N, van den Broek J, Bogte A, Dwars BJ (2013) The incidence and natural course of occult inguinal hernias during TAPP repair: Repair is beneficial. Surg Endosc 27(11):4142–4146 (**3**)
11. Clark JJ, Limm W, Wong LL (2011) What is the likelihood of requiring contralateral inguinal hernia repair after unilateral repair? Am J Surg 202(6):754–757. (**3**)

## Chapter 3: Technical key points in TEP

### Ferdinand Köckerling, Pradeep Chowbey, David Lomanto

**Search terms**: “inguinal hernia”; “femoral hernia”; “total extraperitoneal patch plasty”; “TEP”; “preperitoneal access”; “space creation”; “peritoneal tears”; “complications”.

**Search machines**

In PubMed, Medline, and the Cochran Library as well as in the reference lists of the included studies were searched for relevant studies.

**New publications**

A total of 12 new studies were identified for inclusion. Nine level 1 studies deal with the local extraperitoneal pain treatment during TEP. Two level 3 and one level 4 studies are supplementing the knowledge about the technical key points of direct and indirect sac handling and drainage in TEP repair.

**How should a large direct sac be handled?**

New statements—identical to previous except statement below.

**Table:** 

Level 4	Alternatively to fixation of the extended fascia transversalis to Copper’s ligament the direct inguinal hernia defect can be closed by a pre-tied suture loop (**new statement**).

New recommendations—identical to previous except recommendation below.

**Table:** 

Grade D	As alternative the primary closure of direct inguinal hernia defects with a pre-tied suture loop can be used (**new recommendation**).

**Comments**

Each of the M2 or M3 direct defects, according to the European Hernia Society (EHS), were systematically closed prior to the introduction of the prosthetic mesh [1]. Grasping and inversion of the attenuated transversalis fascia at its apex, using a laparoscopic forceps and plication of the transversalis fascia by placing a tight endoloop of polydioxanone (PDS) at its base. In total, endoloops of PDS were used to close the weakened transversalis fascia in 76 cases (30 M3, 44 M2 and two M1). Only one patient (1.3 %) complained of a residual seroma formation, which was still clinically present at 3 month post-operatively, but was not symptomatic. There were only two minor post-operative complications, which occurred in the same patient and were not related to the endoloop technique. Finally, no patient complained of chronic groin pain and there was no hernia recurrence after a median follow up of 18 months.

**How should a large indirect sac be handled?**

New statement—identical to previous except statement below.

**Table:** 

Level 3	Transection of a large indirect sac does not lead to significant differences in postoperative pain, length of hospital stay and recurrence, but to a significant higher seroma rate (**new statement**).

New recommendation—identical to previous except recommendation below.

**Table:** 

Grade C	A large indirect sac may be ligated proximally and divided distally without the risk of a higher postoperative pain and recurrence rate, but with an increased postoperative seroma rate (**new recommendation**).

**Comments**

520 TEP repairs with indirect inguinal sac were performed in 498 patients. The patients were classified into two groups: the transected sac group with 269 patients (275 cases) and the completely reduced sac group with 230 patients (245 cases) [2]. Statistical analysis between the two groups showed no significant differences in postoperative pain, length of hospital stay, and recurrence, except for postoperative seromas, which were more frequent in the transected sac group (24 of 275) than the completely reduced sac group (6 of 245; *p* = 0.002).

**Should a drain be used after a TEP repair? Should seromas be aspirated?**

New statement—identical to previous except statement below.

**Table:** 

Level 3	Drain after TEP significantly reduces the incidence of seroma formation with increasing the risk of infection or recurrence (**new statement**).

New recommendation—identical to previous except recommendation below.

**Table:** 

Grade C	A closed-suction drain can be used to reduce the risk of seroma formation without increased risk of infection (**new recommendation**).

**Comments**

In 929 patients (1,753 hernias), drain was put in 849 patients (1,607 hernias) and no drain was put in 80 patients (146 hernias) [3]. Follow-up ranged from 9 to 45 months. Seroma formation was significantly lower in the drain group (12/1,607; 0.75 %) compared with the non-drain group (22/146; 15.1 %) (*p* < 0.001). Both the groups were comparable in pain scores, conversion to open, hospital stay, and days taken to return to normal activity and recurrence rates. There was no infection in either group.

**Has extraperitoneal local anesthetic treatment during TEP a positive effect on postoperative pain? New (added) question**

New statement (added)

**Table:** 

Level 1 A	Extraperitoneal bupivancaine treatment during endoscopic TEP inguinal hernioplasty is not more efficacious for the reduction of pain than placebo.

New recommendation (added)

**Table:** 

Grade A	Extraperitoneal bupivacaine treatment during endoscopic TEP inguinal hernia repair for the reduction of postoperative pain should not be performed.

**Comments**

Tong et al. (2013) [4] reviewed eight trials that included a total of 373 patients (5–12). They found no difference between the groups in postoperative pain reduction following endoscopic TEP inguinal hernia repair. The intensity of pain was not significantly different between the bupivacaine treatment group and the control group. No bupivacaine-related complications were reported. They concluded, that extraperitoneal bupivaciane treatment during endoscopic TEP inguinal hernioplasty is not more efficacious for the reduction of postoperative pain than placebo.

### Chapter 3

1. Berney CR (2012) The Endoloop technique for the primary closure of direct inguinal hernia defect during the endoscopic totally extraperitoneal approach Hernia 16:301–305. (**4**)
2. Choi YY, Kim Z, Hur KY (2011) Transection of the hernia sac during laparoscopic totally extraperitoneal inguinal hernioplasty: is it safe and feasible? J Laparoendosc Adv Surg Tech 21:149–152. (**3**)
3. Ismail M, Garg M, Rajagopal M, Garg P (2009) Impact of closed-suction drain in preperitoneal space on the incidence of seroma formation after laparoscopic total extraperitoneal inguinal hernia repair. Surg Laparsc Endosc Percutan Tech 19(3):263–266. (**3**)
4. Tong YS, Wu CC, Bai CH, Lee HC, Liang HH, Kuo LJ, Wei PL, Tam KW (2014) Effect of extraperitoneal bupivacaine analgesia in laparoscopic inguinal hernia repair: a meta-analysis of randomized controlled trials Hernia 18(2):177–183. (**1A**)
5. Abbas MH, Hamade A, Choudhry MN, Hamza N, Nadeem R, Ammori BJ (2010) Infiltration of wounds and extraperitoneal pace with local anesthetic in patients undergoing laparoscopic totally extraperitoneal repair of unilateral inguinal hernias: a randomized double-blind placebo-controlled trial. Scand J Surg 99:18–23. (**1B**)
6. Bar-Dayan A, Natour M, Bar-Zakai B, Zmora O, Shabtai M, Ayalon A, Kuriansky J (2004) Preperitoneal bupivacaine attenuates pain following laparoscopic inguinal hernia repair. Surg Endosc 18:1079–1081. (**1B**)
7. Hon SF, Poon CM, Leong HT, Tang YC (2009) Pre-emptive infiltration of bupivacaine in laparoscopic total extraperitoneal hernioplasty: a randomized controlled trial. Hernia 13:53–56. (**1B**)
8. Kumar S, Joshi M. Chaudhary S (2009) “Dissectalgia” following TEP, a new entity: its recognition and treatment. Results of a prospective randomized controlled trial. Hernia 13:591–596. (**1B**)
9. O’Riordain DS, Kelly P, Horgan PG, Keane FB, Tanner WA (1998) A randomized controlled trial of extraperitoneaal bupivacaine analgesia in laparoscopic hernia repair. Am J Surg 176:254–257. (**1B**)
10. Saff GN, Marks RA, Kuroda M, Rozan JP, Hertz R (1998) Analgesic effect of bupivacaine on extraperitoneal laparoscopic hernia repair. Anesth Analg 87:377–381. (**1B**)
11. Subwongcharoen S, Udompornmongkol V (2010) A randomized control trial of levobupivacaine, bupivacaine versus placebo extraperitoneal infusion in totally extraperitoneal laparoscopic inguinal hernioplasty. J Surg Res 162:279–283. (**1B**)
12. Suvikapakornkul R, Valaivarangkul P, Noiwan P, Phansukphon T (2009) A randomized controlled trial of preperitoneal bupivacaine instillation for reducing pain following laparoscopic inguinal herniorrhaphy. Surg Innov 16:117–123. (**1B**)

## Chapter 4: TEP versus TAPP: which is better?

### Subodh Kumar, Mahesh C. Misra, Virinder K. Bansal, Devanshu Bansal

**Search terms**: TAPP, TEP, TAPP versus TEP, Total Extraperitoneal repair, Trans abdominal Preperitoneal repair, Inguinal hernia

**Search machines**

Cochrane database, PubMed database, Medline database

**New publications**

A total of 200 publications were identified and 11 were used.

New statement—identical to previous except statement below.

**Table:** 

Level 1A	TAPP has a longer hospital stay compared to TEP (**new**).
Level 1B	Potentially serious adverse events are rare after both TAPP and TEP (**stronger evidence**).
	TAPP has a longer operation time compared to TEP (**new**).
Level 2C	TEP has more intra-operative and postoperative surgical complication rate compared to TAPP (**new**).

New recommendations—identical with previous except recommendations below.

**Table:** 

Grade A	Both techniques are acceptable treatment options for inguinal hernia repair and there is sufficient data to conclude that both TAPP and TEP are effective methods of laparoscopic inguinal hernia repair (**stronger evidence**).

**Comments**

*Postoperative/persistent pain*

Bansal et al. [7] randomized 314 patients into two groups (TEP, TAPP) and recorded the postoperative pain score at 6 h, 24 h, 1 week and 6 weeks as well as parenteral analgesic requirement. TAPP group was associated with a significantly higher pain score at 6 h, 24 h, 1 week and 6 weeks. Parenteral analgesic requirement was also found to be significantly higher in the TAPP group. Zanghi et al. [10] prospectively studied 439 patients undergoing TEP or TAPP repair. Postoperative pain score was higher in the TAPP group on 1, 7, 30 and 90 days postoperatively.

*Visceral injury*

In the RCT done by Bansal et al. [7], no major intraoperative complications with no hollow viscus, bladder injury, or major vascular injury were seen. None of the patients in either group had any life-threatening complications during the postoperative period in form of deep vein thrombosis (DVT) and pulmonary embolism (PE) or myocardial infarction (MI).

*Deep infection*

No incidence of deep infection were seen postoperatively in level 1 and 2 studies [1–11].

*Port site hernia*

No incidence of port site hernia were seen postoperatively in level 1 and 2 studies [1–11].

*Seroma*

Bansal et al. [7] found a significantly higher incidence of postoperative seroma in the TEP repair group. Postoperative seroma were managed by observation only.

*Scrotal edema*

Bansal et al. [7] found a significantly higher incidence of postoperative scrotal edema in the TAPP repair group.

*Operative time*

TAPP repair group was associated with a significantly longer operative time compared to the TEP group [7]. In the population based study by Gass et al. [11], TEP repair was associated with a significantly longer operating time compared to TAPP group.

*Hospital stay*

In the meta analysis by Bracale et al. [1], there was a significantly longer postoperative hospital stay in the TAPP group. Bansal et al. [7] did not find any significant difference in the postoperative hospital stay between TAPP and TEP repair. Gass et al. [11] also found a significantly longer hospital stay in the TAPP group.

*Conversion rate*

Bansal et al. [7] had a single conversion in the TEP group, because the anatomy could not be defined due to adhesions between peritoneum, posterior rectus sheath, and abdominal wall fascia, which lead to peritoneal laceration leading to conversion. However, the repair could be accomplished after conversion to TAPP. Gass et al. [11] found that unadjusted and risk-adjusted analyses of conversion rates revealed significantly higher rates for the TEP group, as is reflected by a high odds ratio.

*Complication rate*

Gass et al. [11] found that patients undergoing TEP had a statistically significant increased rate of intraoperative complications and postoperative surgical complications. General postoperative complications were not statistically different between the two methods.

*Recurrence rate*

Bansal et al. [7] had one recurrence in TAPP group (0.3 %), where mesh was found to have migrated into the dilated internal inguinal ring at reoperation and forming part of the sac. No recurrences were seen in the TEP repair group.

*Overall satisfaction*

No difference in the overall satisfaction was found between TEP and TAPP in level 1 and 2 studies [1–11].

*Quality of life*

In the study by Bansal et al. [7], both the TEP and TAPP groups showed significant improvement in quality of life from the preoperative period to 3 months postoperatively. The TEP group showed significant improvement in all domains, whereas the TAPP group showed significant improvement in all domains except those of vitality and social functions. However, both groups were comparable postoperatively in terms of quality of life. No previous studies have compared quality of life after TEP versus TAPP repair.

## References (in parentheses graduation of evidence)

1. Bracale U, Melillo P, Pignata G, Salvo E, Rovani M, Merola G, Pecchia L (2012) Which is the best laparoscopic approach for inguinal hernia repair: TEP or TAPP? A systematic review of the literature with a network meta-analysis. Surg Endosc 26:3355–3366. (**1A**)
2. Antoniou S, Antoniou G, Bartsch D, Fendrich V, Koch O, Pointner R, Granderath F (2013) Transabdominal preperitoneal versus totally extraperitoneal repair of inguinal hernia: a meta-analysis of randomized studies. Am J Surg 206:245–252. (**1A**)
3. Gong K, Zhang N, Lu Y, Zhu B, Zhang Z, Du D, Zhao X, Jiang H (2011) Comparison of the open tension-free mesh-plug, transabdominal preperitoneal (TAPP), and totally extraperitoneal (TEP) laparoscopic techniques for primary unilateral inguinal hernia repair: a prospective randomized controlled trial. Surg Endosc 25:234–239. (**1B**)
4. Mesci A, Korkmaz B, Dinckan A, Colak T, Balci N, Ogunc G (2012) Comparison of the open tension-free mesh-plug, transabdominal preperitoneal (TAPP), and totally extraperitoneal (TEP) laparoscopic techniques for primary unilateral inguinal hernia repair: a prospective randomized controlled trial. Surg Today 42:157–163. (**1B**)
5. Hamza Y, Gabr E, Hammadi H, Khalil R (2010) Four-arm randomized trial comparing laparoscopic and open hernia repairs. Int J Surg 8: 25–28. (**1B**)
6. Zhu Q, Mao Z, Yu B, Jin J, Zheng M, Li J (2009) Effects of Persistent CO_2_ insufflation during different laparoscopic inguinal hernioplasty: a prospective, randomized, controlled study. J Laparoendosc Adv Surg Tech A 19:611–614. (**1B**)
7. Bansal VK, Misra MC, Babu D, Victor J, Kumar S, Sagar R, Rajeshwari S, Krishna A, Rewari V (2013) A prospective, randomized comparison of long-term outcomes: chronic groin pain and quality of life following totally extraperitoneal (TEP) and transabdominal preperitoneal (TAPP) laparoscopic inguinal hernia repair. Surg Endosc 27:2373–2382. (**1B**)
8. Krishna A, Misra MC, Bansal VK, Kumar S, Rajeshwari S, Chabra A (2012) Laparoscopic inguinal hernia repair: transabdominal preperitoneal (TAPP) versus totally extraperitoneal (TEP) approach: a prospective randomized controlled trial. Surg Endosc 26:639–649. (**1B**)
9. Belyansky I, Tsirline V, Klima D, Walters A, Lincourt A, Heniford T (2011) Prospective, comparative study of postoperative quality of life in TEP, TAPP, and modified lichtenstein repairs. Ann Surg 254:709–715. (**2B**)
10. Zanghì A, Di Vita M, Lo Menzo E, Castorina S, Cavallaro AS, Piccolo G, Grosso G, Cappellani A (2011) Multicentric evaluation by verbal rate scale and EuroQoL-5D of early and late post-operative pain after TAPP and TEP procedures with mechanical fixation for bilateral inguinal hernias. Ann Ital Chir 82:437–442. (**2B**)
11. Gass M, Banz V, Rosella L, Adamina M, Candinas D, Guller U (2012) TAPP or TEP? Population-based analysis of prospective data on 4,552 patients undergoing endoscopic inguinal hernia repair. World J Surg 36:2782–2786. (**2C**)

## Chapter 5: Endoscopic/laparoscopic surgery in complicated hernias: feasibility, risks, and benefit

### George Ferzli, Michel Timoney

**Search terms**: ‘‘Scrotal hernia’’; ‘‘Hernias with large defects’’, “Recurrent inguinal hernia”, “Femoral hernia”, “Incarcerated hernia”, “Occult inguinal hernia”, “Strangulated hernia”, “Synchronous inguinal hernia

**Search machines**

PubMed

**New publications**

No new publications found.

**TAPP and TEP for scrotal hernia repair**

New statements—identical to previous except statement below.

**Table:** 

Level 3	TEP inguinal-scrotal hernia repair remains an advantageous approach during the difficult scrotal hernia that requires “conversion” to an open repair, because the pre-peritoneal dissection performed laparoscopically allows for reduction of the hernia and optimal mesh placement once the hernia repair has been converted and is performed from the anterior approach (**new**).

New recommendations—identical to previous except recommendation below.

**Table:** 

Grade C	TEP approach for the large, difficult scrotal hernia may serve as an adjunct to dissection and definition of the pre-peritoneal space allowing for easier hernia and mesh placement once the case is “converted” to open repair (**new**).

**Comments**

Ferzli et al. [1] reviewed their experience with 1,890 TEP hernia repairs. Ninety-four large scrotal hernias were identified of which, nine cases (9.5 %) required conversion to an open procedure due to an incarcerated and indurated omentum. Six of these (6.4 %) underwent a combined laparoscopic and open repair with good results and no recurrence at 6 months. They conclude that a combined laparoscopic and open approach can greatly assist in the visualization and dissection of the preperitoneal space, thereby facilitating reduction of the hernia and placement of the mesh.

Siow et al. [2] retrospectively reviewed their experience with TAPP in the treatment of incarcerated scrotal hernias. They were able to successfully treat 20 patients using either a pure TAPP technique or TAPP combined with a limited open technique.

**TAPP for incarcerated and strangulated inguinal hernia**

No new statements or recommendations.

**TEP for incarcerated and strangulated inguinal hernia**

New statement—identical to previous except statement below.

**Table:** 

Level 3	Laparoscopic hernia repair for incarcerated inguinal hernia has been successfully and safely performed in the pediatric population (**new**).

New recommendations—identical to previous except recommendations below.

**Table:** 

Grade C	Laparoscopic hernia repair for incarcerated inguinal hernia may be successfully and safely performed in the pediatric population by surgeons with laparoscopic expertise (**new**).

**Comments**

Nah et al. [3] performed a retrospective study of pediatric patients with incarcerated inguinal hernias and found a trend toward fewer complications in the group whose repair was performed laparoscopically rather than open, although this was not statistically significant. They also found a higher statistically significant incidence of contralateral hernias that were repaired at the time of repair of the incarcerated hernia.

Esposito et al. [4] reviewed their experience with 601 children who underwent laparoscopic inguinal hernia repair 46 (7.6 %) of whom presented with incarceration. The authors were able to successfully treat these patients with laparoscopic repair with a recurrence rate of 4.3 %.

Chan et al. [5] reviewed their experience with laparoscopic approach to the incarcerated pediatric inguinal hernia repair. They were able to safely and successfully treat 16 patients with incarcerated hernias using laparoscopy. Choi et al. [6] conducted a retrospective analysis of 945 patients who underwent TEP repair of their inguinal hernia and 66 had an incarcerated hernia. There was no difference in outcome between the incarcerated and reducible groups but operative times were longer and seroma formation was greater in the incarcerated group.

Yang et al. [7] retrospectively reviewed 188 patients who underwent emergency surgical repair of strangulated groin hernias; 57 received laparoscopic and 131 received open repairs. They found that more laparotomies were performed in the open group (19 vs. 0), the wound infection rate was significantly higher in the open group (12 vs. 0), and the mean hospital stay was shorter in the laparoscopic group (4.39 vs. 7.34 days).

**TAPP and TEP for incarcerated femoral hernia Statements**

No new statements or recommendations.

**Comments**

Ginesta et al. [8] published a case report of successful TEP hernioplasty combined with laparoscopic assisted intestinal resection for a strangulated Richter femoral hernia.

**Laparoscopic inguinal hernia repair in the setting of peritonitis and bowel necrosis**

No new statements or recommendations.

**TAPP for recurrent inguinal hernia**

No new statements or recommendations.

**TEP for recurrent inguinal hernia**

No new statements or recommendations.

**Comments**

Demetrashvili et al. [9] performed a randomized prospective study comparing open versus TAPP repair for recurrent inguinal hernia. Twenty eight patients were assigned to the Lichtenstein repair technique and 24 to TAPP repair. Results were equivalent in terms of operative time, recurrence and chronic pain. The TAPP patients had significantly less pain in the postoperative period and, faster recovery.

Shah et al. [10] found no difference in complication rate in their retrospective review of 172 patients who underwent either open versus laparoscopic inguinal hernia repair for recurrent inguinal hernia. They did find a significantly lower incidence of re-recurrence in the laparoscopic group. Sevonius et al. [11] reviewed the Swedish hernia registry and found that the risk of reoperation for re-recurrence in 19,582 hernia repairs for recurrent hernia is significantly reduced if the laparoscopic or open pre-peritoneal repair were used for the repair of the recurrence (*p* < 0.001). Bignell et al. [12] prospectively studied 120 patients who underwent TAPP inguinal hernia repair versus open hernia repair. They demonstrated a slightly lower severity of chronic groin pain after laparoscopic inguinal hernia repair for bilateral and recurrent inguinal hernias versus open repair but with no significant improvement in quality of life. Yildiz et al. [13] reviewed 26 male pediatric patients who underwent laparoscopic repair of recurrent hernia. Thirteen were treated with laparoscopic surgery (with Schier’s intracorporeal “N” suture closure) and 13 with open surgery (with high ligation technique). They found a statistically shorter length of the operation time in laparoscopic repair group.

**TAPP / TEP inguinal hernia repair after failed TAPP / TEP**

*No new* statements or recommendations.

**Comments**

van den Heuvel and Dwars [14] reviewed 2,594 TAPP inguinal hernia repairs (TAPP). Of these, 53 repairs were attempted for recurrent hernias after a previous posterior repair in 51 patients. Two repairs had to be converted to an open technique. One case resulted in ligation of the vas deferens. Four patients developed port site hernias. There were no serious postoperative events. At follow-up (mean of 70 months) no recurrences were found.

Uchida et al. [15] retrospectively reviewed 28 patients who underwent TEP repair of a contralateral inguinal hernia out of 215 who had undergone previous TEP inguinal hernia repair. Complications in this group were few. Three patients required conversion to an anterior approach and, in four, the inferior epigastric artery and vein were divided.

**TAPP and TEP repair in patient after previous transabdominal radical prostatectomy**

No new statements or recommendations

**Pitfalls of TAPP and TEP repair for recurrent inguinal hernia**

No new statements or recommendations.

**TAPP and TEP repair and the occult synchronous hernias**

New statements—identical to previous except statement below.

**Table:** 

Level 4	Women are at increased risk of having an occult synchronous femoral hernia (**New**).

New recommendation—identical to previous except recommendation below.

**Table:** 

Grade C	When performing inguinal hernia repair in women, extra effort should be undertaken to reveal and treat occult synchronous femoral hernia (**New**).

**Comments**

Putnis et al. [16] performed a retrospective review of 362 patients who underwent 484 TEP inguinal hernia repairs. They found a total of 18 cases of synchronous femoral hernias with a statistically higher incidence of femoral hernia in females (37 %) compared to males (3 %) (*p* < 0.001). They suggest that all women presenting with an inguinal hernia also have a formal assessment of the femoral canal.

Henrikson et al. [17] looked at 461 patients undergoing laparoscopic hernia repair for the incidence of occult synchronous femoral hernia. They found a significantly higher incidence of unsuspected femoral hernia in patients undergoing repair for recurrence [23/250, 9.2 %) compared to the group undergoing primary repair (8/211, 3.8 %), *p* = 0.02. Furthermore, 38.1 % of women operated on for a recurrent inguinal hernia, presented with an unsuspected femoral hernia at surgery as opposed to 6.6 % of the men, *p* = 0.003.

Dulucq et al. [18] prospectively performed 337 laparoscopic inguinal hernia repairs in 263 patients. These patients were all assessed for occult concomitant hernia. 44 unexpected hernias were encountered and repaired with minimal complication: 6 Spiegelian hernias, 19 obturator hernias and 19 femoral hernias. Nah et al. [3] performed a retrospective study of pediatric patients with incarcerated inguinal hernias and found a trend toward fewer complication in the group whose repair was performed laparoscopically rather than open, although this was not statistically significant. They also found a higher statistically incidence of contralateral hernias which were repaired at the time of repair of the incarcerated hernia.

## References (in parentheses graduation of evidence)

1. Ferzli GS, Rim S, Edwards ED (2013) Combined laparoscopic and open extraperitoneal approach to scrotal hernias. Hernia 17(2):223–228. (**3**)
2. Siow SL, Mahendran HA, Hardin M, Chea CH, NikAzim NA (2013) Laparoscopic transabdominal approach and its modified technique for incarcerated scrotal hernias. Asian J Surg 36(2):64–68. (**3**)
3. Nah SA, Giacomello L, Eaton S, de Coppi P, Curry JI, Drake DP, Kiely EM, Pierro A (2011) Surgical repair of incarcerated inguinal hernia in children: laparoscopic or open? Eur J Pediatr Surg 21(1):8–11. (**3**)
4. Esposito C, Turial S, Alicchio F, Enders J, Castagnetti M, Krause K, Settimi A, Schier F (2013) Laparoscopic repair of incarcerated inguinal hernia. A safe and effective procedure to adopt in children. Hernia 17(2):235–23. (**3**)
5. Chan KW, Lee KH, Tam YH, Sihoe JD, Cheung ST, Mou JW (2011) Laparoscopic inguinal hernia repair by the hook method in emergency setting in children presenting with incarcerated inguinal hernia. J Pediatr Surg 46 (10):1970–1973. (**3**)
6. Choi YY, Kim Z, Hur KY (2011) Laparoscopic total extraperitoneal repair for incarcerated inguinal hernia. J Korean Surg Soc 80(6):426–430. (**3**)
7. Yang GP, Chan CT, Lai EC, Chan OC, Tang CN, Li MK (2012) Laparoscopic versus open repair for strangulated groin hernias: 188 cases over 4 years. Asian J Endosc Surg 5(3):131–137. (**3**)
8. Ginestà C, Saavedra-Perez D, Valentini M, Vidal O, Benarroch G, García-Valdecasas JC (2013) Total extraperitoneal (TEP) hernioplasty with intestinal resection assisted by laparoscopy for a strangulated richter femoral hernia surgical laparoscopy. Endosc Percutan Tech 23(3): 334–336. (**5**)
9. Demetrashvili Z, Qerqadze V, Kamkamidze G, Topchishvili G, Lagvilava L, Chartholani T, Archvadze V (2011) Comparison of lichtenstein and laparoscopic transabdominal preperitoneal repair of recurrent inguinal hernias. Int Surg 96(3):233–238. (**1B**)
10. Shah NR, Mikami DJ, Cook C, Manilchuk A, Hodges C, Memark VR, Volckmann ET, Hall CR, Steinberg S, Needleman B, Hazey JW, Melvin WS, Narula VK (2011) A comparison of outcomes between open and laparoscopic surgical repair of recurrent inguinal hernias. Surg Endosc 25(7):2330–2337. (**3**)
11. Sevonius D, Gunnarsson U, Nordin P, Nilsson E, Sandblom G (2011) Recurrent groin hernia surgery. BJS 98(10):1489–1494. (**2C**)
12. Bignell M, Partridge G, Mahon D, Rhodes M (2012). Prospective randomized trial of laparoscopic (transabdominal preperitoneal-TAPP) versus open (mesh) repair for bilateral and recurrent inguinal hernia: incidence of chronic groin pain and impact on quality of life: results of 10 year follow-up. Hernia 16(6): 635–640. (**2B**)
13. Yildiz A, Çelebi S, Akin M, Karadağ ÇA, Sever N, Erginel B, Dokucu AI (2012) Laparoscopic herniorraphy: a better approach for recurrent hernia in boys? Pediatr Surg Int 28(5):449–453. (**3**)
14. van den Heuvel B, Dwars BJ (2013) Repeated laparoscopic treatment of recurrent inguinal hernias after previous posterior repair. Surg Endosc 27(3):795–800. (**3**)
15. Uchida H, Matsumoto T, Endo Y, Kusumoto T, Muto Y, Kitano S (2011) Repeat laparoscopic totally extraperitoneal hernia repair after primary laparoscopic totally extraperitoneal hernia repair for inguinal hernia. J Laparoendosc Adv Surg Tech A 21(3):233–235. (**3**)
16. Putnis S, Wong A, Berney C (2011) Synchronous femoral hernias diagnosed during endoscopic inguinal hernia repair. Surg Endosc 25: 3752–3754. (**3**)
17. Henriksen NA, Thorup J, Jorgensen LN (2012) Unsuspected femoral hernia in patients with a preoperative diagnosis of recurrent inguinal hernia. Hernia 16(4):381–385. (**2B**)
18. Dulucq JL, Wintringer P, Mahajna A (2011) Occult hernias detected by laparoscopic totally extra-peritoneal inguinal hernia repair: a prospective study. Hernia 15(4):399–402. (**2B**)

## Chapter 6: Mesh size and recurrence

### Thue Bisgaard, Jacob Rosenberg

**Search terms**: “Hernia, Inguinal [MESH] (“size” or “recurrence”), “clinical trial”, randomized controlled—“meta-analysis”.

**Search machines**

PubMed and the Cochrane Database of Systematic Reviews specialized register and reference lists of the included studies were searched for studies for potential inclusion.

**New publications**

A total of 81 new studies were identified (compared with former literature search covering 1966 to January 2009) and none of them were relevant.

No new statements or recommendations.

## References (in parentheses graduation of evidence)

No references.

## Chapter 7: Heavy or light weight mesh in TAPP and TEP—functional outcome and quality of life

### Dirk Weyhe, F. Koeckerling, Uwe Klinge

**Search terms**: “TAPP” AND “mesh”, TEP AND “mesh”, “Biocompatibility” AND “mesh”, “groin pain” AND “mesh”, “inguinal hernia” AND “mesh”, “Quality of life” AND “mesh”, “azoospermia” AND “mesh”, “sperm-motility” AND “mesh”

**Search machines**

Pubmed, Medline, and Cochrane Library.

**New publications**

*TAPP* In total, *n* = 26 hits were found from February 2009–October 2013. Excluding *n* = 2 (review a.o.), *n* = 23 publications were classified according to the evidence criteria. The result was *n* = 3/23 articles fulfilled the criteria of Level IB (13 %) based on Oxford hierarchy of evidence [1–3]. However, these papers are disregarded by reason that they are not comparing mesh types in TAPP.

*TEP* The TEP search resulted in *n* = 34 hits. Excluding *n* = 3 articles (listed in TAPP search), *n* = 1 (3 %) article correlate to level 1B [4]. In a one-year follow up midterm results are described in this RCT.

Overall *n* = 3 meta-analysis are available [5–7]. Since the publication of the IEHS Guidelines in 2011, *n* = 3 prospectively randomized trials and *n* = 1 registry study have been published concerning azoospermia [8–11].

New statements—identical to previous except statement below.

**Table:** 

Level 1 A	The statistical significance that lighter meshes with larger pores results in improvement of quality of life *is not consistent* in recently published meta-analyses. Subset analysis revealed no higher risk of recurrence after using lightweight meshes in laparoscopic inguinal hernia repair (**New**).
Level 2B	The middle- and long-term results of prospective studies in men do not support the hypothesis that bilateral inguinal hernia repair with alloplastic mesh prosthesis causes male infertility or decreasing the sperm motility (**New**).

New recommendations—identical to previous except recommendation below.

**Table:** 

Grade B	A monofilament implant with a pore size of at least 1.0–1.5 mm (usually meaning low-weight) consisting of a minimum tensile strength in all directions (including subsequent tearing force) of 16 N/cm appeared to be most advantageous; however, this assumption mainly summarizes personal and published clinical and experimental experiences (**stronger evidence**).
	The application of large pore polypropylene meshes in endoscopic hernia repair is harmless concerning azoospermia and should therefore further used (**New**).

**Comments**

**Fig. 1:**
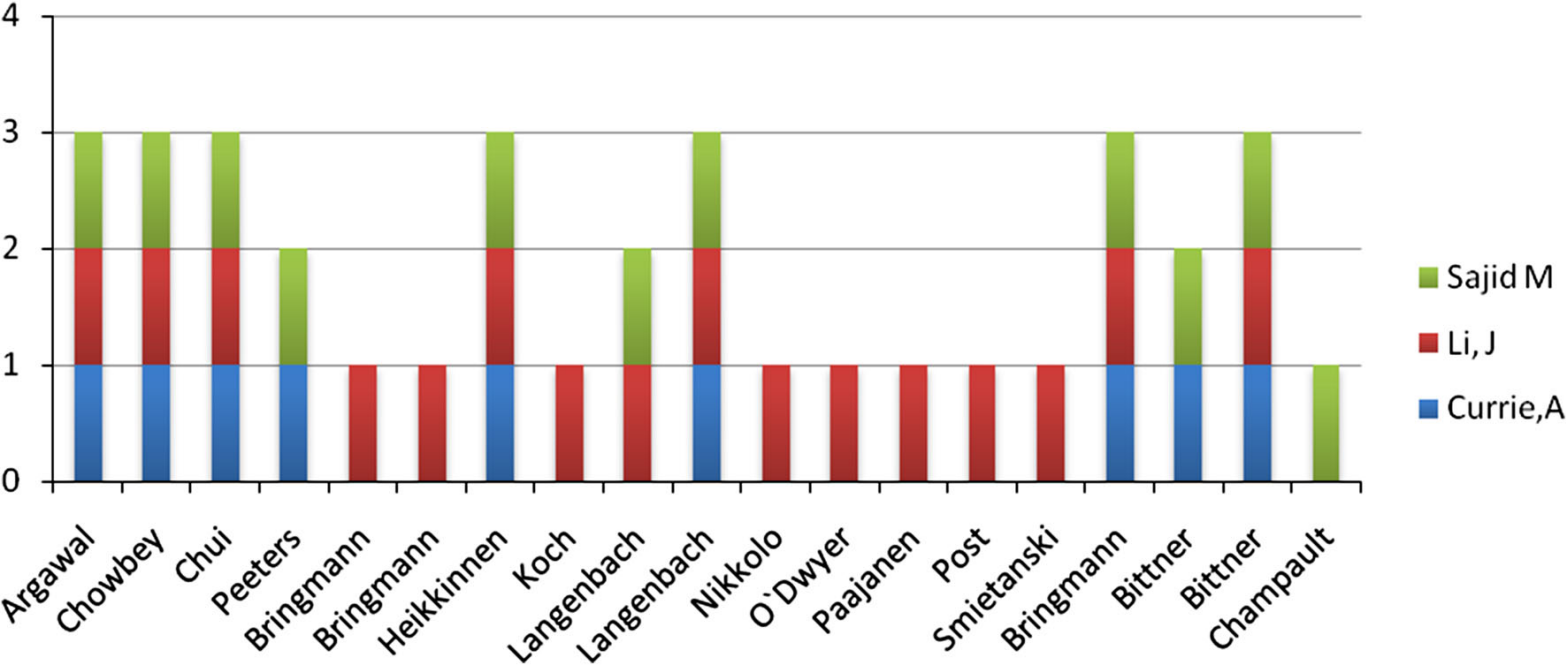
Accordance of included RCT’s in published meta-analysis from Sajid, Li and Currie [4, 14–30]

A clear recommendation cannot be made based on currently published RCT’s even if level 1A evidence is available. Two of three meta-analyses found no significant differences in terms of early postoperative pain, recurrence rate or return to work [5, 7]. The reduced incidence of chronic groin pain is only in one meta-analysis [6] significantly lower after LM implantation. Li et al. evaluated a publication bias by using Egger’s test but mixed different techniques in hernia repair. Regardless of the addition of non-randomized but controlled trails, there is no difference in the development of chronic groin pain within 6 months between both mesh types. Interestingly, out of a total of 16 RCT’s which are used for the structured review by Currie et al. [5], Li et al. [7] and Sajid et al. [6], only *n* = 6 were cited in the three published meta-analysis (Fig. 1). In addition only Sajid [6] includes data from Champault [12] and independently from the discussion if Champault study is prospective randomized or not, it influenced substantial this meta-analysis. Therefore the value is arguable. However, based on a slight trend to improved quality of life after using large pore and so called lightweight meshes, the authors upgrades the existing recommendation from Grade D to Grade B even if the present meta-analysis are not statistical consistent.

The lack of consistency of the results of published RCT”s suggests that, on one hand, the mesh-choice only slightly influence the clinical outcome and, on the other hand, the classification in heavy and light meshes does not allow sufficient differentiation. On this account, a modified implant classification with primary regard to the local scarring formation than the implants weight should be done in future to allow better comparability of RCT’s [13]. Concerning azoospermia as an important parameter regarding quality of life, a Belgian prospective study showed significant early postoperative sperm-motility disorders in the light-mesh group and could not be noticed in long-term examinations [9, 10]. A Swedish registry study compared patients receiving meshes with such without mesh implantation [11]. This study could exclude, independently of the mesh type, a higher risk of infertility.

## References (in parentheses graduation of evidence)

1. Krishna A, Misra MC, Bansal VK, Kumar S, Rajeshwari S, Chabra A (2012) Laparoscopic inguinal hernia repair: transabdominal preperitoneal (TAPP) versus totally extraperitoneal (TEP) approach: a prospective randomized controlled trial. Surg Endosc 26(3):639–649. (**1B**)
2. Gong K, Zhang N, Lu Y, Zhu B, Zhang Z, Du D, Zhao X, Jiang H (2011) Comparison of the open tension-free mesh-plug, transabdominal preperitoneal (TAPP), and totally extraperitoneal (TEP) laparoscopic techniques for primary unilateral inguinal hernia repair: a prospective randomized controlled trial. Surg Endosc 25(1):234–239. (**1B**)
3. Hamza Y, Gabr E, Hammadi H, Khalil R (2010) Four-arm randomized trial comparing laparoscopic and open hernia repairs. Int J Surg 8(1):25–28. (**1B**)
4. Chui LB, Ng WT, Sze YS, Yuen KS, Wong YT, Kong CK (2010) Prospective, randomized, controlled trial comparing lightweight versus heavyweight mesh in chronic pain incidence after TEP repair of bilateral inguinal hernia. Surg Endosc 24(11):2735–2738. (**1B**)
5. Currie A, Andrew H, Tonsi A, Hurley PR, Taribagil S (2012) Lightweight versus heavyweight mesh in laparoscopic inguinal hernia repair: a meta-analysis. Surg Endosc 26(8):2126–2133. (**1A**)
6. Sajid MS, Kalra L, Parampalli U, Sains PS, Baig MK (2013) A systematic review and meta-analysis evaluating the effectiveness of lightweight mesh against heavyweight mesh in influencing the incidence of chronic groin pain following laparoscopic inguinal hernia repair. Am J Surg 205(6):726–736. (**1A**)
7. Li J, Ji Z, Cheng T (2012) Lightweight versus heavyweight in inguinal hernia repair: a meta-analysis. Hernia 16(5):529–39. (**1B**)
8. Skawran S, Weyhe D, Schmitz B, Belyaev O, Bauer KH (2011) Bilateral endoscopic total extraperitoneal (TEP) inguinal hernia repair does not induce obstructive azoospermia: data of a retrospective and prospective trial. World J Surg 35(7):1643–1648. (**2C**)
9. Peeters E, Spiessens C, Oyen R, De Wever L, Vanderschueren D, Penninckx F, Miserez M (2014) Sperm motility after laparoscopic inguinal hernia repair with lightweight meshes: 3-year follow-up of a randomised clinical trial. Hernia 18(3):361–367. (**2B**)
10. Peeters E, Spiessens C, Oyen R, De Wever L, Vanderschueren D, Penninckx F, Miserez M (2010) Laparoscopic inguinal hernia repair in men with lightweight meshes may significantly impair sperm motility: a randomized controlled trial. Ann Surg 252(2):240–246. (**2B**)
11. Hallén M, Sandblom G, Nordin P, Gunnarsson U, Kvist U, Westerdahl J, (2011) Male infertility after mesh hernia repair: a prospective study. Surgery 149(2):179–184. (**2C**)
12. Champault G, Bernard C, Rizk N, Polliand C (2007) Inguinal hernia repair: the choice of prosthesis outweighs that of technique. Hernia 11:125–124. (**3**)
13. Klinge U, Klosterhalfen B (2012) Modified classification of surgical meshes for hernia repair based on the analyses of 1,000 explanted meshes. Hernia 16(3):251–258. (**2C**)
14. Agarwal BB, Agarwal KA, Mahajan KC (2009) Prospective double-blind randomized controlled study comparing heavy- and lightweight polypropylene mesh in totally extraperitoneal repair or inguinal hernia: early results. Surg Endosc 23:242–247. (**2B**)
15. Chowbey PK, Barg N, Sharma A, Khullar R, Soni V, Baijal M, Mittal T (2010) Prospective randomized clinical trial comparing lightweight mesh and heavyweight polypropylene mesh in endoscopic totally extraperitoneal groin hernia repair. Surg Endosc 24:3073–3079. (**2B**)
16. Chui LB, Ng WT, Sze YS, Yuen KS, Wong YT, Kong CK (2010) Prospective, randomized, controlled trial comparing lightweight versus heavyweight mesh in chronic pain incidence after TEP repair of bilateral inguinal hernia. Surg Endosc 24(11):2735–2738. (**1B**)
17. Peeters E, Spiessens C, Oyen R, De Wever L, Vanderschueren D, Penninckx F, Miserez M (2010) Laparoscopic inguinal hernia repair in men with lightweight meshes may significantly impair sperm motility: a randomized controlled trial. Ann Surg 252:240–246. (**2B**)
18. Bringman S, Heikkinen TJ, Wollert S, Österberg J, Smedberg S, Granlund H, Ramel S, Felländer G, Anderberg B (2004) Early results of a single-blinded, randomized, controlled, Internet-based multicenter trial comparing Prolene and Vypro II mesh in Lichtenstein hernioplasty. Hemia 8:127–134. (**2B**)
19. Bringman S, Wollert S, Österberg J, Smedberg-S, Granlund H, Fellinder G, Heikkinen T (2005) One year results of a randomized controlled multi-centre study comparing Prolene and Vypro Il-mesh in Lichtenstein hernioplasty. Hernia 9:223–227. (**2B**)
20. Heikkinen T, Wollert S, Österberg J, Smedberg S, Bringman S (2006) Early results of a randomized trial comparing Prolene and Vypro Il-mesh in endoscopic extraperitoneal inguinal hernia repair (TEP) of recurrent unilateral hernias. Hernia 10:34–40. (**2B**)
21. Koch A, Bringman S, Myrelid P, Smeds S, Kald A (2008) Randomized clinical trial of groin hernia repair with titanium coated lightweight mesh compared with standard polypropylene mesh. Br J Surg 95(10):1226–1231. (**2B**)
22. Langenbach MR, Schmidt J, Zirngibl H (2006) Comparison of biomaterials: three meshes and TAPP for inguinal hernia. Surg Endosc 20:1511–1517. (**2B**)
23. Langenbach MR, Schmidt J, Ubrig B, Zirngibl H (2008) Sixty-month follow-up after endoscopic inguinal hernia repair with three types of mesh: a prospective randomized trial. Surg Endosc 22:1790–1797. (**2B**)
24. Nikkolo C, Lepner U, Mumrste M, Vaasna T, Seepter H, Tikk T (2010) Randomized clinical trial comparing lightweight mesh with heavyweight mesh for inguinal hernioplasty. Hernia 14:253–258. (**2B**)
25. O’Dwyer PJ, Kingsnorth AN, Molloy RG, Smal1 PK, Lammers B, Horeyseck G (2005) Randomized clinical trial assessing impact of a lightweight or heavyweight mesh on chronic pain after inguinal hernia repair. Br JSurg 92(2):166–170. (**2B**)
26. Paajanen H (2007) A single-surgeon randomized trial comparing three composite meshes on chronic pain after Lichtenstein hernia repair in local anesthesia. Hernia 11(4):335–339. (**2B**)
27. Post S, Weiss B, Willer M, Neufang T, Lorenz D (2004) Randomized clinical trial of lightweight composite mesh for Lichtenstein inguinal hernia repair. Br J Surg 91(1):44–48. (**2B**)
28. Smietanski M, for the Polish Hernia Study Group (2008) Randomized clinical trial comparing a polypropylene with a poliglecaprone and polypropylene composite mesh for inguinal hernioplasty. Br J Surg 95:1462–1468. (**2B**)
29. Bittner R, Leibl BJ, Kraft B, Schwarz J (2011) One-year results of a prospective, randomized clinical trial comparing four meshes in laparoscopic inguinal hernia repair (TAPP). Hernia 15:503–510. (**2B**)
30. Bittner R, Schmedt CG, Leibl BJ, Schwarz J (2011) Early postoperative and one year results of a randomized controlled trial comparing the impact of extralight titanized polypropylene mesh and traditional heavyweight polypropylene mesh on pain and seroma production in laparoscopic hernia repair (TAPP). World J Surg 35(8):1791–1797. (**2B**)

## Chapter 8: Slitting or not slitting of mesh—does it influence outcome?

### Thue Bisgaard, Jacob Rosenberg

**Search terms**: Hernia, Inguinal [MESH] (“cutting” or “slit”), “clinical trial”, “randomized controlled”—“meta-analysis”.

**Search machines**

PubMed and the Cochrane Database of Systematic Reviews specialized register and reference lists of the included studies were searched for studies for potential inclusion.

**Number of publications**

A total of 176 new studies were identified (compared with former literature search covering 1966 to January 2009) and two of them were relevant.

New statements—identical to previous except statement below.

**Table:** 

Level 1	Cutting a slit in the mesh to allow the structures of the funicel to pass does not compromise testicular perfusion and testicular volume (**New**).

New recommendations—identical to previous except recommendations below.

**Table:** 

Grade B	Based on available evidence we recommend not to cut a slit in the mesh although cutting does not compromise testis perfusion (**New**).

**Comments**

We identified one new randomized trial [1]. In this trial [1] 40 patients undergoing TEP were randomized to a slit or no slit. Doppler ultrasound was performed preoperatively, day 5 and after 6 months. There were no significant differences in testicular perfusion and volume.

Finally, one case–control study [2] with a retrospective design compared 78 patients undergoing TEP with a slit mesh with 300 patients undergoing TEP with a no-slit mesh. Number of patients included was not based on a power analysis. Patients had a 12 × 15 cm polypropylene mesh. Clinical recurrences were seen in 0.6 % in the slit group and in 6 % in the no-slit group (*p* < 0.01). Follow-up after 3 years was either with telephone interview or clinical examination and the study quality was questionable since significant bias may have been involved in patient selection for slit versus no-slit.

There is no convincing evidence to support use of a slit or to use no-slit in the mesh for laparoscopic inguinal hernia repair. One study found some of the recurrences to be associated with insufficient closure of the mesh slit. This could argue against slitting the mesh. We routinely do not cut a slit in the mesh as it does not bring any technical advantage for the surgeon or better clinical results for the patient.

## References (in parentheses graduation of evidence)

1. Celik AS, Memmi N, Celebi F, Guzey D, Celik A, Kaplan R, Oncu M (2009) Impact of slit and nonslit mesh technique on testicular perfusion and volume in the early and late postoperative period of the totally extraperitoneal preperitoneal technique in patients with inguinal hernia. Am J Surg 198:287–291. (**1B**)
2. Domniz N, Perry ZH, Lantsberg L, Avinoach E, Mizrahi S, Kirshtein B (2011) Slit versus non-slit mesh placement in total extraperitoneal inguinal hernia repair. World J Surg 35:2382–2386. (**3**)

## Chapter 9: Mesh fixation modalities: is there an association with acute or chronic pain?

### René H. Fortelny, Wolfgang Reinpold, Agneta Montgomery

**Search terms**: ‘‘Surgical Mesh (MeSH)’’ AND ‘‘Surgical fixation device’’ (MeSH) AND ‘‘Inguinal Hernia’’ (MeSH); ‘‘fixation AND mesh AND TEP’’; ‘‘fixation AND mesh AND TAPP’’; ‘‘TAPP AND pain’’; ‘‘TEP AND pain’’; ‘‘groin hernia AND pain’’; ‘‘inguinal hernia AND pain’’; “Randomized control trial” AND “fixation AND hernia”.

**Search machines**

PubMed and the Cochrane Database of Systematic Reviews specialized register and reference lists of the included studies were search for studies for potential inclusion.

**New publications**

A total of 10 new studies were identified as Level 1. Four studies on non-fixation versus mechanical fixation were identified. Three were meta-analysis [1–3] and the last one by Sajid et al. [3] reported on eight RCTs that was used for the analyses. One RCT was published after this meta-analysis and was included in this analysis [4]. Five studies on glue fixation versus mechanical fixation were identified. Two were meta-analysis [5, 6] and the last one by Sajid [6] et al. reported on 5 RCTs and were used in this analyses. Another five new RCTs [8–11] have been published since and have been included in this analysis.

New statements—identical to previous except recommendations below.

**Table:** 

Level 1A	Fixation and non-fixation of the mesh in TEP are associated with equally risk of postoperative pain or recurrence (**New**).
Level 1B	Fibrin glue fixation is associated with less chronic pain than stapling.

New recommendations—identical to previous except recommendations below.

**Table:** 

Grade A	If TEP technique is used, non-fixation has to be considered in all types of inguinal hernias except large direct defects (MIII, EHS classification) (**stronger recommendation**).
Grade B	In case of TAPP repair non-fixation should be considered in types LI, II, and MI, II hernias (EHS classification).
	For fixation, fibrin glue should be considered to minimize the risk of acute postoperative pain (**modified recommendations**).

**Comments**

Sajid et al. reported in the meta-analysis on no difference between non-fixation versus mechanical fixation for both early (overall effect *Z* = 0.75 *p* = 0.45) and chronic pain (*Z* = 0.43 *p* = 0.67) [3]. The RCT of Garg et al. [4], published after this meta-analysis, confirmed the same results. This evidence is the background for the new statement Level 1A.

Sajid et al. [3] reported in their second meta-analysis no difference between glue fixation and mechanical fixation for early pain (*Z* = 1.27, *p* = 0.20). There was a significant difference for chronic pain (*Z* = 3.27, *p* = 0.001) [6]. Three studies reported on early pain after the meta-analysis [8–10]. They all concluded that early pain was significantly less in the glue group. Four studies reported on chronic pain after the meta-analysis demonstrating no difference between glue and mechanical fixation [8–10]. This led to the decision to exclude the former recommendation to consider fibrin glue to minimize the risk of chronic pain.

Concerning the use of self-fixating meshes up to now only one randomized controlled trial comparing fixation by fibrin glue versus micro-hooks is published 2012 without any significant difference concerning postoperative pain in a follow up of 3 months [11]. For information Cochrane Colorectal Cancer Group specialized register reported an on-going meta-analysis of mesh fixation techniques for laparoscopic inguinal hernia repair [12].

## References (in parentheses graduation of evidence)

1. Tam KW, Liang HH, Chai CY (2010) Outcomes of staple fixation of mesh versus nonfixation in laparoscopic total extraperitoneal inguinal repair: a meta-analysis of randomized controlled trials. World J Surg 34(12):3065–3074. (**1A**)
2. Teng YJ, Pan SM, Liu YL, Yang KH, Zhang YC, Tian JH, Han JX (2011) A meta-analysis of randomized controlled trials of fixation versus nonfixation of mesh in laparoscopic total extraperitoneal inguinal hernia repair. Surg Endosc 25(9):2849–2858. (**1A**)
3. Sajid MS, Ladwa N, Kalra L, Hutson K, Sains P, Baig MK (2012) A meta-analysis examining the use of tacker fixation versus no-fixation of mesh in laparoscopic inguinal hernia repair. Int J Surg 10(5):224–231. (**1A**)
4. Garg P, Nair S, Shereef M, Thakur JD, Nain N, Menon GR, Ismail M (2011) Mesh fixation compared to nonfixation in total extraperitoneal inguinal hernia repair: a randomized controlled trial in a rural center in India. Surg Endosc 25(10):3300–3306. (**1B**)
5. Kaul A, Hutfless S, Le H, Hamed SA, Tymitz K, Nguyen H, Marohn MR (2012) Staple versus fibrin glue fixation in laparoscopic total extraperitoneal repair of inguinal hernia: a systematic review and meta-analysis Surg Endosc 26:1269–1278. (**1A**)
6. Sajid MS, Ladwa N, Kalra L, McFall M, Baig MK, Sains P (2013) A meta-analysis examining the use of tacker mesh fixation versus glue mesh fixation in laparoscopic inguinal hernia repair. Am J Surg 206(1):103–111. (**1A**)
7. Fortelny RH, Petter-Puchner AH, May C, Jaksch W, Benesch T, Khakpour Z, Redl H, Glaser KS (2012) The impact of atraumatic fibrin sealant vs. staple mesh fixation in TAPP hernia repair on chronic pain and quality of life: results of a randomized controlled study. Surg Endosc 26(1):249–254. (**1B**)
8. Brügger L, Bloesch M, Ipaktchi R, Kurmann A, Candinas D, Beldi G (2012) Objective hypoesthesia and pain after transabdominal preperitoneal hernioplasty: a prospective, randomized study comparing tissue adhesive versus spiral tacks. Surg Endosc 26(4):1079–1085. (**1B**)
9. Subwongcharoen S, Ruksakul K (2013) A randomized controlled trial of staple fixation versus N-butyl-2-cyanoacrylate fixation in laparoscopic inguinal hernia repair. J Med Assoc Thai. 96 Suppl 3:8–13. (**2B**)
10. Tolver MA, Rosenberg J, Juul P, Bisgaard T (2013) Randomized clinical trial of fibrin glue versus tacked fixation in laparoscopic groin hernia repair. Surg Endosc 27(8):2727–2733. (**1B**)
11. Cambal M, Zonca P, Hrbaty B (2012) Comparison of self-gripping mesh with mesh fixation with fibrin-glue in laparoscopic hernia repair (TAPP). Bratisl Lek Listy 113(2):103–107. (**1B**)
12. Dickinson K, McCormack K, Scott N, Fawole A, White C, Grant AM. Mesh fixation techniques for laparoscopic inguinal hernia repair in adults. Cochrane Database Syst Rev 2011, Issue 1. Art. No.: CD008954. doi: 10.1002/14651858.CD008954. (**1A**)

## Chapter 10: Risk factors and prevention of acute and chronic pain in TAPP and TEP

### Wolfgang Reinpold

**Search terms**: “TEP” and “pain”; “TAPP” and “pain”; “groin hernia” and “pain”; “inguinal hernia” and “pain”; “randomized controlled trial” and “pain” and “hernia”.

**Search machines**

Pubmed, Medline, Embase, British Journal of Surgery database, Science Citation Index, and the Cochrane database.

**New publications**

A total of 13 new studies were identified as Level 1. There is one new systematic review comparing open versus TEP and TAPP for acute and chronic pain [1] and one systematic review comparing TEP and TAPP for acute pain [2].

New statements—identical to previous except statements below.

**Table:** 

Level 1A	There is no difference of chronic pain after TEP and TAPP (**stronger evidence**).
	Fixation and non fixation of the mesh in TEP are associated with equally risk of postoperative pain (see chapter “Fixation”) (**new**).
Level 1B	Fibrin glue fixation is associated with less chronic pain than stapling (see chapter “Fixation”) (**new**).
Level 2A	Age below median (40–50 years) is a risk factor for acute pain (**stronger evidence**).
	Age below median (40–50 years) is a risk factor for chronic pain (**stronger evidence**).
	Severe acute postoperative pain is a risk factor for chronic pain (**stronger evidence**).

New recommendations—identical to previous except recommendations below.

**Table:** 

Grade A	If TEP technique is used non fixation has to be considered in all types of inguinal hernias except large defects (L III, MIII; EHS classification; see chapter “Fixation”) (**new**).
Grade B	In case of TAPP repair non fixation should be considered in types LI, LII, MI, MII hernias (EHS classification, see Chapter “Fixation”) (**new**).

**Comments**

Four new RCT compared TEP and TAPP for pain [3–6] of which three analyzed only chronic pain [3, 5, 6]. While there was no difference for chronic pain, two RCT [3, 6] reported less acute pain after TEP. There were identified 9 new RCT [4, 5, 7–13] including 3,780 patients comparing open repair with TEP/TAPP repair. Two of these trials analyzed only chronic pain. All seven studies reported less acute pain after TAPP/TEP. Eight trials found significant less chronic pain after TAPP/TEP. One systematic review [2] identified young age as risk factor for acute pain and one RCT reported more chronic pain in younger patients. One systematic review [2] and one RCT [7] identified severe acute postoperative pain as risk factor for chronic pain.

## References (in parentheses graduation of evidence)

1. Bracale U, Melillo P, Pignata G, Di Salvo E, Rovani M, Merola G, Pecchia L (2012) Which is the best laparoscopic approach for inguinal hernia repair: TEP or TAPP? A systematic review of the literature with a network meta-analysis. Surg Endosc 26:3355–3366. (**1A**)
2. Tolver MA, Rosenberg J, Bisgaard T (2012) Early pain after laparoscopic inguinal hernia repair. A qualitative systematic review. Acta Anaesthesiol Scand 56(5):549–557. (**1A**)
3. Bansal VK, Misra MC, Babu D, Victor J, Kumar S, Sagar R, Rajeshwari S, Krishna A, Rewari V (2013) A prospective, randomized comparison of long-term outcomes: chronic groin pain and quality of life following totally extraperitoneal (TEP) and transabdominal preperitoneal (TAPP) laparoscopic inguinal hernia repair. Surg Endosc 27(7):2373–2382. (**1B**)
4. Hamza Y, Gabr E, Hammadi H, Khalil R (2010) Four-arm randomized trial comparing laparoscopic and open hernia repairs. Int J Surg 8:25–28. (**1B**)
5. Gong K, Zhang N, Lu Y, Zhu B, Zhang Z, Du D, Zhao X, Jiang H (2011) Comparison of the open tension-free mesh-plug, transabdominal preperitoneal (TAPP), and totally extraperitoneal (TEP) laparoscopic techniques for primary unilateral inguinal hernia repair: a prospective randomized controlled trial. Surg Endosc 25:234–239. (**1B**)
6. Krishna A, Misra MC, Bansal VK, Kumar S, Rajeshwari S, Chabra A (2012) Laparoscopic inguinal hernia repair: transabdominal preperitoneal (TAPP) versus totally extraperitoneal (TEP) approach: a prospective randomized controlled trial. Surg Endosc 26:639–649. (**1B**)
7. Singh AN, Bansal VK, Misra MC, Kumar S, Rajeshwari S, Kumar A, Sagar R, Kumar A (2012) Testicular functions, chronic groin pain, and quality of life after laparoscopic and open mesh repair of inguinal hernia: a prospective randomized controlled trial. Surg Endosc 26(5):1304–1317. (**1B**)
8. Dahlstrand U, Sandblom G, Ljungdahl M, Wollert S, Gunnarsson U (2013) TEP under general anesthesia is superior to Lichtenstein under local anesthesia in terms of pain 6 weeks after surgery: results from a randomized clinical trial. Surg Endosc 27:3632–3638. (**1B**)
9. Aigner F, Augustin F, Kaufmann C, Schlager A, Ulmer H, Pratschke J, Schmid T (2014) Prospective, randomized-controlled trial comparing postoperative pain after plug and patch open repair with totally extraperitoneal inguinal hernia repair. Hernia 18(2):237–242. (**1B**)
10. Bektaş H, Bilsel Y, Ersöz F, Sarı S, Mutlu T, Arıkan S, Kaygusuz A (2011) Comparison of totally extraperitoneal technique and darn plication of primary inguinal hernia. J Laparoendosc Adv Surg Tech A 21:583–588. (**1B**)
11. Eker HH, Langeveld HR, Klitsie PJ, van’t Riet M, Stassen LP, Weidema WF, Steyerberg EW, Lange JF, Bonjer HJ, Jeekel J (2012) Randomized clinical trial of total extraperitoneal inguinal hernioplasty vs Lichtenstein repair: a long-term follow-up study. Arch Surg 147:256–260. (**1B**)
12. Langeveld HR, van’t Riet M, Weidema WF, Stassen LP, Steyerberg EW, Lange J, Bonjer HJ, Jeekel J (2010) Total extraperitoneal inguinal hernia repair compared with Lichtenstein (the LEVEL-Trial): a randomized controlled trial. Ann Surg 251:819–824. (**1B**)
13. Eklund A, Montgomery A, Bergkvist L, Rudberg C; Swedish Multicentre Trial of Inguinal Hernia Repair by Laparoscopy (SMIL) study group (2010) Chronic pain 5 years after randomized comparison of laparoscopic and Lichtenstein inguinal hernia repair. Br J Surg 97:600–608. (**1B**)

## Chapter 11: Urogenital complications associated with TAPP and TEP

### Robert J. Fitzgibbons

**Search terms**: Laparoscopic inguinal herniorrhaphy, urinary complications, testicular complications, spermatic cord complications, infertility, sexual dysfunction.

**Search machines**

Pubmed, Medline.

**Bladder perforation**

No new statements or recommendations.

**Mesh erosion into the bladder**

No new statements or recommendations.

**Comments**

Mesh erosion into the bladder after LIH is rare, probably occurring in well less than 1 % of cases. The literature dealing with this complication is made up almost exclusively of case reports and therefore the complication is under reported so that the exact incidence is not known [1].

**Urinary retention**

No new statements or recommendations.

**Comment**

One reference is confirming previous statement [2].

**Urinary infection**

No new statements or recommendations

**Miscellaneous cord and testicular problems**

No new statements or recommendations.

**Ischemic orchitis /testicular atrophy**

No new statements or recommendations.

**Sexual Dysfunction**

No new statements or recommendations.

**Comments**

Post herniorrhaphy inguinal, genital or ejaculatory pain occurs in a small percentage of men after groin hernia repair. In a Danish study comprised of men undergoing a laparoscopic inguinal hernia repair who were registered in the Danish Hernia Database, dysejaculation occurred in 3.1 % [3]. Some pain in the groin or genitals was reported during sexual activity in 10.9 % and in 2.4 % the impaired sexual activity was moderate or severe. The incidence is probably underestimated because of the reluctance of patients to discuss their sexual function. The cause is not completely understood. There is no consistently effective therapy but alpha receptor blockers to decrease contractility of the Vas and neurolytic agents such as Pregabalin have been tried. Erectile dysfunction is another complication which men occasionally report after inguinal herniorrhaphy but its direct relationship makes little anatomical sense and the incidence is unknown

**Infertility**

**New statement**

**Table:** 

Level 2B	Inguinal hernia repair with mesh is not associated with an increased risk of, or clinically important risk for, male infertility. (**new**).

**New recommendation**

**Table:** 

Grade B	Groin hernia repair using mesh techniques may continue to be performed without major concern about the risk for male infertility. (**new**).

**Comments**

Although animal studies have suggested a strong correlation between mesh inguinal hernia repairs and structural damage to elements of the spermatic cord and testicle [4], this has not translated into a clinically significant infertility rate after open or laparoscopic inguinal hernia repair [4–6]. A concern that the light weight meshes might have a greater adverse effect on sperm motility, seen 1 year after total extraperitoneal inguinal hernia repair (TEP) in one study [7], could not be confirmed at 3 years follow up [8].

## References (in parentheses graduation of evidence)

1. Hamouda A, Kennedy J, Grant N, Nigam A, Karanjia N (2010) Mesh erosion into the urinary bladder following laparoscopic inguinal hernia repair; is this the tip of the iceberg? Hernia 14(3): 317–319. (**4**)
2. Sivasankaran MV, Pham T, Divino CM.(2014) Incidence and risk factors for urinary retention following laparoscopic inguinal hernia repair. Am J Surg 207(2):288–292. (**4**)
3. Bischoff JM, Linderoth G, Aasvang EK, Werner MU, Kehlet H (2012) Dysejaculation after laparoscopic inguinal herniorrhaphy: a nationwide questionnaire study. Surg Endosc 26(4):979–983. (**2C**)
4. Tekatli H, Schouten N, van Dalen T, Burgmans I, Smakman N (2012) Mechanism, assessment, and incidence of male infertility after inguinal hernia surgery: a review of the preclinical and clinical literature. Am J Surg 204(4):503–509. (**2A**)
5. Skawran S, Weyhe D, Schmitz B, Belyaev O, Bauer KH (2011) Bilateral endoscopic total extraperitoneal (TEP) inguinal hernia repair does not induce obstructive azoospermia: Data of a retrospective and prospective trial. World J Surg 35:1643–1648. (**2B**)
6. Hallen M, Westerdahl J, Nordin P, Gunnarsson U, Sandblom G (2012) Mesh hernia repair and male infertility: a retrospective register study. (**2C**)
7. Peeters E, Spiessens C, Oyen R, De Wever L, Vanderschueren D, Penninckx F, Miserez M (2010) Laparoscopic inguinal hernia repair in men with lightweight meshes may significantly impair sperm motility. Ann Surg 252:240–246. (**1B**)
8. Peeters E, Spiessens C, Oyen R, De Wever L, Vanderschueren D, Penninckx F, Miserez M (2014) Sperm motility after laparoscopic inguinal hernia repair with lightweight meshes: 3-year follow-up of a randomised clinical trial. Hernia 18(3):361–367. (**1B**)

## Chapter 12: Intraperitoneal onlay mesh (IPOM) for inguinal hernia repair—still a therapeutic option?

### Kevin L. Grimes, Kirpal Singh, Maurice E. Arregui

**Search terms**: “IPOM”; “intraperitoneal onlay mesh”; “inguinal hernia” AND “intraperitoneal” AND “onlay” AND “mesh”.

**Search machines**

PubMed; Medline.

**New publications**

PubMed search yielded 61 and Medline search yielded 43 publications, which were screened for relevance. There was no level 1 or level 2 publications during the search period.

No new statements or recommendations

## References (in parentheses graduation of evidence)

- No new references.

## Chapter 13: Role for open preperitoneal mesh placement in the era of endo/laparoscopic inguinal hernia repair

### Kevin L. Grimes, Kirpal Singh, Maurice E. Arregui

**Search terms**: “open preperitoneal hernia repair”; “laparoscopic inguinal hernia repair”; “TAPP” AND “preperitoneal” AND “hernia repair”; “TEP” AND “preperitoneal” AND “hernia repair”; “preperitoneal” AND “hernia” AND “repair”.

**Search machines**

PubMed; Medline.

**New publications**

Pubmed search yielded 117 and Medline search yielded 145 publications, which were screened for relevance. Three studies during the search period were Level 1 or 2.

New statements—identical to previous except statements below.

**Table:** 

Level 1B	Minimally invasive open approaches (i.e., Kugel) may offer a cost advantage over laparoscopic approaches. (**new**).

No new recommendations

**Comments**

Recent literature does not support a change to previous recommendations. Bender, et al. [1] randomized 40 patients to either Kugel or TEP repair of unilateral hernias. There were no significant differences in operative time, length of stay, return to activity, or serum inflammatory markers. Cost was US$546 lower with Kugel. Hamza, et al. [2] randomized 100 patients to open pre-peritoneal, Lichtenstein, TAPP, or TEP. Laparoscopic approaches were associated with less pain and faster return to activity. Ozmen et al. [3] compared flow dynamics and cross-sectional area of femoral vessels following either TEP or Stoppa procedures. There was no evidence of DVT or significant changes in flow characteristics as a result of mesh placement in either technique.

## References (in parentheses graduation of evidence)

1. Bender O, Balci FL, Yuney E, Saglam F, Ozdenkaya Y, Sari YS (2009) Systemic inflammatory response after Kugel versus laparoscopic groin hernia repair: a prospective randomized trial. Surg Endosc 23:2657–2661. (**1B**)
2. Hamza Y, Gabr E, Hammadi H, Khalil R (2010) Four-arm randomized trial comparing laparoscopic and open hernia repairs. Int J Surg 8:25–28. (**1B**)
3. Ozmen M, Zulfikaroglu B, Ozalp N, Moran M, Soydinc P, Ziraman I (2010) Femoral vessel blood flow dynamics following totally extraperitoneal vs Stoppa procedure in bilateral inguinal hernias. Am J Surg 199:741–745. (**1B**)

## Chapter 14: Single port surgery or reduced ports in endoscopic/laparoscopic hernia repair (New chapter)

### Davide Lomanto

**Search terms**: Inguinal Hernia, Laparoscopy/methods, Surgical instruments, Single port, Single port access, Reduced port surgery, Surgical technique, Laparoscopic surgery, Minimally invasive surgery.

**Search machines**

Pubmed, Embase and Medline.

**Number of publications**

24 Papers are relevant: 5 level 2B; 19 level 4.

Statements

**Table:** 

**Level 2B**	Single port laparoscopic hernia repair is a safe and feasible alternative to traditional multiport technique although has not been showed to be superior or more effective.
	Single port laparoscopic hernia repair may offer a better cosmetic outcome and patient’s satisfaction.
	Single port laparoscopic hernia repair has no increased risk compared with standard multiport technique.
	Homemade ports, as an alternative to commercially available ports, provides a feasible and safe alternatives

**Recommendations**

**Table:** 

Grade B	Single port laparoscopic inguinal hernia repair is safe and feasible alternative options to conventional laparoscopy in selected cases but further RCTs are needed.
	Both TAPP and TEP can be performed with equal results in selected cases.

**Comments**

In the last few years, minimally invasive surgery has continued to develop by further reducing surgical aggression and scars hence Natural Orifice Transluminal Endoscopic Surgery (NOTES) came into light. This new approach created a lot of enthusiasm but still several issues and challenges have arisen and need to be resolved before a full clinical acceptance [1–3]. While improving on these procedures, the idea of reducing the number and size of ports, so-called single incision access surgery came into limelight. In the beginning by using multiple fascial punctures and later using dedicated devices that were ad hoc developed and marketed. Through a small wound incision between 1.5 and 2.5 cm, the single port device can be inserted and allow multiple access for telescope and instrumentations to carried out the surgery. Early reports of different procedures have been published and the cosmetic advantage offered by the single port endo-laparoscopic surgery (SPES) make this approach attractive option for patients who require additional benefit of cosmesis. Further clinical studies involving large series of patients, are needed to confirm the benefits and advantages of SPES over standard procedure. Some case reports and cohort studies have been published on single port inguinal hernia repair [4–30]. Two RCT Trials has been published recently from high volume centers in which safety, efficacy and improved cosmesis was confirmed with an overall outcome similar to standard technique [31–32].

## References (in parentheses graduation of evidence)

1. Romanelli JR, Earle DB (2009) Single-port laparoscopic surgery: an overview. Surg Endosc 23:1419–1427. (**5**)
2. Allemann P, Schafer M, Demartines N (2010) Critical appraisal of single port access cholecystectomy. Br J Surg 97:1476–1480. (**2C**)
3. Tracy CR, Raman JD, Cadeddu JA, Rane A (2008) Laparoendoscopic single-site surgery in urology: where have we been and where are we heading? Nat Clin Pract Urol 5:561–568. (**5**)
4. Goo TT,Goel R, Lawenko M, Lomanto D (2010) Laparoscopic transabdominal preperitoneal (TAPP) hernia repair via a single port. Surg Laparosc Endosc Percutan Tech 20:389–390. (**4**)
5. Jacob BP, Tong W, Reiner M, Vine A, Katz LB (2009) Single incision total extraperitoneal (one SITE) laparoscopic inguinal hernia repair using a single access port device. Hernia 13:571–572. (**4**)
6. Buckley FP III, Vassaur H, Monsivais S, Sharp NE, Jupiter D, Watson R, Eckford J (2014) Comparison of outcomes for single-incision laparoscopic inguinal herniorrhaphy and traditional three-port laparoscopic herniorrhaphy at a single institution. Surg Endosc 28(1): 30–35. (**2B**)
7. Yilmaz H, Alptekin H (2013) Single-incision laparoscopic transabdominal preperitoneal herniorrhaphy for bilateral inguinal hernias using conventional instruments. Surg Laparosc Endosc Percutan Tech 23(3):320–323. (**4**)
8. Takayama S, Nakai N, Sakamoto M, Takeyama H (2014) Single-incision laparoscopic herniorrhaphy for inguinal hernia repair. Surg Today 44(3):513–516. (**4**)
9. Pesta W, Kurpiewski W, Luba M, Szynkarczuk R, Grabysa R (2012) Single incision laparoscopic surgery transabdominal pre-peritoneal hernia repair—case report. Wideochir Inne Tech Malo Inwazyjne 7(2):137–139. (**4**)
10. Fuentes MB, Goel R, Lee-Ong AC, Cabrera EB, Lawenko M, Lopez-Gutierrez J, Lomanto D (2013) Single-port endo-laparoscopic surgery (SPES) for totally extraperitoneal inguinal hernia: a critical appraisal of the chopstick repair. Hernia 17(2):217–221. (**2B**)
11. Soon Y, Yip E, Onida S, Mangat H (2012) Single-port hernia repair: a prospective cohort of 102 patients. Hernia 16(4):393–396. (**2B**)
12. Kim JH, Park SM, Kim JJ, Lee YS (2011) Initial experience of single port laparoscopic totally extraperitoneal hernia repair: nearly-scarless inguinal hernia repair. J Korean Surg Soc 81(5):339–343. (**2B**)
13. Shih TY, Wen KC, Lin KY, Uen YH (2012) Transumbilical, single-port, totally extraperitoneal, laparoscopic inguinal hernia repair using a homemade port and a conventional instrument: an initial experience. J Laparoendosc Adv Surg Tech A 22(2):162–164. (**4**)
14. Tran H (2011) Robotic single-port hernia surgery. JSLS 15(3):309–314. (**4**)
15. Tran H (2011) Safety and efficacy of single incision laparoscopic surgery for total extraperitoneal inguinal hernia repair. JSLS 15(1):47–52. (**2B**)
16. Tai HC, Lin CD, Chung SD, Chueh SC, Tsai YC, Yang SS (2011) A comparative study of standard versus laparoendoscopic single-site surgery (LESS) totally extraperitoneal (TEP) inguinal hernia repair. Surg Endosc 25(9):2879–2883. (**2B**)
17. Lee YS, Kim JH, Hong TH, Lee IK, Oh ST, Kim JG, Badakhanian R (2011) Transumbilical single-port laparoscopic transabdominal preperitoneal repair of inguinal hernia: initial experience of single institute. Surg Laparosc Endosc Percutan Tech 21(3):199–202. (**4**)
18. Chung SD, Huang CY, Wang SM, Hung SF, Tsai YC, Chueh SC, Yu HJ (2011) Laparoendoscopic single-site totally extraperitoneal adult inguinal hernia repair: initial 100 patients. Surg Endosc 25(11):3579–3583. (**2B**)
19. Do M, Liatsikos E, Beatty J, Haefner T, Dunn I, Kallidonis P, Stolzenburg JU (2011) Laparoendoscopic single-site extraperitoneal inguinal hernia repair: initial experience in 10 patients. J Endourol 25(6):963–968. (**4**)
20. Kucuk C (2011) Single-incision laparoscopic transabdominal preperitoneal herniorrhaphy for recurrent inguinal hernias: preliminary surgical results. Surg Endosc 25 (10):3228–34. (**4**)
21. Tai HC, Ho CH, Tsai YC (2011) Laparoendoscopic single-site surgery: adult hernia mesh repair with homemade single port. Surg Laparosc Endosc Percutan Tech 21(1):42–45. (**4**)
22. Goo TT, Goel R, Lawenko M, Lomanto D (2010) Laparoscopic transabdominal preperitoneal (TAPP) hernia repair via a single port. Surg Laparosc Endosc Percutan Tech 20(6):389–390. (**4**)
23. Roy P, De A (2010) Single-incision laparoscopic TAPP mesh hernioplasty using conventional instruments: an evolving technique. Langenbecks Arch Surg 395(8):1157–1160. (**4**)
24. He K, Chen H, Ding R, Hua R, Yao Q (2011) Single incision laparoscopic totally extraperitoneal inguinal hernia repair. Hernia 15(4):451–453. (**4**)
25. Macdonald ER, Ahmed I (2010) “Scarless” laparoscopic TAPP inguinal hernia repair using a single port. Surgeon 8(3):179–181. (**4**)
26. Surgit O (2010) Single-incision Laparoscopic surgery for total extraperitoneal repair of inguinal hernias in 23 patients. Surg Laparosc Endosc Percutan Tech 20(2):114–118. (**4**)
27. Agrawal S, Shaw A, Soon Y (2010) Single-port laparoscopic totally extraperitoneal inguinal hernia repair with the TriPort system: initial experience. Surg Endosc 24(4):952–956. (**4**)
28. Menenakos C, Kilian M, Hartmann J (2010) Single-port access in laparoscopic bilateral inguinal hernia repair: first clinical report of a novel technique. Hernia 14(3):309–312. (**4**)
29. Cugura JF, Kirac I, Kulis T, Janković J, Beslin MB (2008) First case of single incision laparoscopic surgery for totally extraperitoneal inguinal hernia repair. Acta Clin Croat 47(4):249–252. (**4**)
30. Kroh M, Rosenblatt S (2009) Single-port, laparoscopic cholecystectomy and inguinal hernia repair: first clinical report of a new device. J Laparoendosc Adv Surg Tech A 19(2):215–217. (**4**)
31. Tran H, Turingan I, Tran K, et al. (2014) Potential benefits of single-port compared to multiport laparoscopic inguinal herniorraphy: a prospective randomized controlled study. Hernia (May 14). [Epub ahead of print]. (**1B**)
32. Wijerathne S, Agarwal N, Ramzi A, Lomanto D (2014) A prospective randomized controlled trial to compare single-port endo-laparoscopic surgery versus conventional TEP inguinal hernia repair. Surg Endosc (Jun 6) [Epub ahead of print]. (**1B**)

## Chapter 15: Convalescence after hernia surgery (New chapter)

### Hartmut Buhck

**Search terms**: Hernia, inguinal/SU, treatment outcome, recurrence, convalescence, activities of daily living, work, exercise, weight, heavy, lifting, strain.

**Search machines**

MEDLINE, Cochrane Library, Embase, manual search for pertinent articles in published article and book references

**Time period of search**

End of search period Dec 31st, 2013; no restriction with regard to the begin of the search period due to the overall very limited amount of high-level evidence.

**Introduction**

Since intra-abdominal pressure plays a triggering—albeit not causative—role in inguinal hernia development, the avoidance of physical strain has been traditionally recommended after surgical repair. However, intra-abdominal pressure—the putative link between physical strain and recurrence—has not been objectively established as a risk factor for recurrence yet [1].

Recommendations for periods of physical inactivity after groin hernia repair are very variable and typically rather long (4–6 weeks) [2, 3], and mostly just expert opinions rather than the result of systematic research [4]. Presently available guidelines are based on cohort or case–control studies of low evidence [5]. There are a precious few reports of clinical trials on this issue [6], and reliable, evidence-based recommendations for a requirement of physical inactivity after hernia repair are notably absent [7, 8]. Since the most current guideline [5] recommends some caution in patients doing heavy lifting (“Probably a limitation on heavy weight lifting for 2–3 weeks is enough”) without specifying either the probability or the threshold of “heavy”, physicians may decide to err on the side of caution rather than recommend a too-early return to work.

Therefore, one of the key outcome parameters of hernia surgery is based on arbitrary decisions rather than representing an objective feature of procedural quality, diminishing the informative value of the published results. Moreover, there is insufficient evidence to support the surgeon while making a decision of quite substantial impact: False recommendations may lead to unnecessary recurrences with potentially hazardous consequences for the patient [9, 10] on the one or economic penalties for patient and/or society due to unnecessary vocational downtime on the other hand.

The issue of convalescence is of particular importance in the context of endoscopic hernia repair since reduced postoperative pain and shorter periods of recovery are some of the key advantages of this approach. Due to the relative paucity of pertinent published evidence, the literature search for the issue of convalescence was not limited in terms of publication dates and evidence levels.

A meticulous analysis of all published evidence yielded no indication for a relationship between postoperative physical strain and risk of hernia recurrence. The only randomized controlled trials (RCTs) on the issue were performed in the same hospital in Nottingham and published about 30 years ago [11–13]. After an initial 3-week period of physical inactivity, patients received different recommendations for the ensuing time (immediate full occupational and recreational activity vs. activity according to the GP’s recommendation [11, 12] or reduced strain for an additional 3 months [13], respectively). GPs recommended extended periods of restrained activity, and immediate full workload had no adverse effects. On the contrary, the only recurrences observed by Taylor et al. [13] occurred after the extended reduced activity.

In a number of retrospective studies, patients were advised to resume full physical activities early after the operation, and did so without any negative impact on the recurrence rates, which were well under 1 % [14, 15]. In addition, a sizeable number of RCTs compared different hernia repair techniques and employed return to work and/or activities of daily living (ADL) as endpoints; these trials uniformly failed to demonstrate a relationship between early rehabilitation on the one and hernia recurrence on the other hand [8]. On the contrary, there are some studies showing the opposite tendency: In a prospective comparison of different recommendations for convalescence presented by Bay-Nielsen et al. [16], three groups of patients treated with the Liechtenstein procedure received the following advice:

- immediate full activity without strain limits (*n* = 1,069).
- reduced activity for 3–4 weeks (*n* = 1,306) or
- no specific recommendations (8,297 reference patients from the Danish Hernia Database).

There were no significant differences between groups in terms of hernia recurrence, but alas, the recurrence rate in the first group was only half as high (0.7 %) as in the others (1.6 and 1.4 %, respectively). This difference is hardly attributable to the early resumption of activity but probably reflects a better standard of care in the study center; however, it underlines the absence of an increased recurrence risk due to early rehabilitation when the surgical procedure was faultless. The importance of the latter point is emphasized by a relatively broad spread of recurrence incidence between centers that suggests procedure-related prognostic factors; for instance, the German Quality Assurance Office [17] and the European Hernia Society [5] reported recurrence rates of as low as 0 % and as high as 19 % in contemporary series surveys.

In conclusion, groin hernia recurrence is obviously surgeon- and not burden-related. Obviously, the following recommendations only address the issues that are specific for groin hernia repair; general rules and precautions of convalescence after ambulatory or day-case surgery certainly apply to those patients as well.

**Is post-surgery physical strain related to groin hernia recurrence?**

Statements

**Table:** 

Level 1B	There is no evidence for an increase in recurrence risk due to physical strain (including heavy lifting) after groin hernia surgery irrespective of the method of surgery.
Level 3	Immediate return to work (within 1–3 days) is not associated with hernia recurrence.
	Immediate resumption of activity of daily living (ADL) (within 1–3 days) is not associated with hernia recurrence.
	Short convalescence is not associated with a higher recurrence risk, and some studies even show an inverse relation

**Recommendations**

**Table:** 

Grade B	Patients should be actively assured that physical activity of any kind does not jeopardize the stability of groin hernia repair.
	Patients should be encouraged to resume work and ADL after 1 day.

**What are the limiting factors for the resumption of work and physical activities after groin hernia repair?**

Statements

**Table:** 

Level 2A	Pain is an important limiting factor for the resumption of work and physical activities after groin hernia repair.
Level 3	Patients’ attitude toward convalescence is heavily influenced by their surgeons’ recommendation.
	Return to work is heavily influenced by the type of sick-leave compensation.

**Recommendations**

**Table:** 

Grade C	Effective pain control is a prerequisite of early return to work and ADL.
Grade B	Patients should be counseled with regard to availability and side effects of analgesics.

**Comments**

The published literature shows a wide variety of periods of sick-leave and return to ADL; the difference between the lowest and highest published figures amounts approximately to a factor of 10 (return to work 5–50 days, resumption of ADL 3–30 days) [8]. This clearly demonstrates the absence of objective criteria for recommendation, and a broad spread like that can hardly reflect the consideration of recurrence risk alone.

Careful analysis of the limiting factors for return to work and ADL shows three issues of relevance:

- *Within series* of patients with identical recommendations by the surgeon, pain is the single most important reason stated for extended periods of inactivity [16, 18–20].
- *Between* series, there are two important factors
  - recommendation given by the surgeon (and the resulting expectation of the patient) [19, 21–23].
  - type and generosity of sick-leave compensation [10, 24, 25].

An American case–control comparison between patients covered by “worker’s compensation” or private health insurance, respectively [24], graphically corroborates the importance of socio-economic circumstances: not only did the former group return much later to work (33.5 ± 4.6 vs. 12.6 ± 2.3 days), but it also reported persistent pain for a sixfold period (111.0 ± 42.2 vs. 17.8 ± 7.9 days).

Whereas the latter point cannot be easily influenced by the surgeon, the two former points show clear and broad avenues to shorter periods of convalescence: Clear recommendation of very short periods (1–3 days) of physical inactivity and generous analgesics prescription, obviously under consideration of patient- and work-specific side effects and risks.

The importance of the patient’s expectation—that is easily influenced by the surgeon—is confirmed by the observation that dispositional pessimism as a personality trait significantly delays return to work after hernia repair [26]. The fact that early postoperative pain is an important precursor of chronic pain after hernia repair [27] corroborates the recommendation of a generous analgesics prescription regimen. This issue is of particular relevance since there are clues that chronic pain after hernia repair—a relatively frequent residuum [28]—is promoted by early resumption of physical activities in patients who experience early postoperative pain [29].

**What period of physical inactivity, if any, is recommended after groin hernia repair?**

No specific period of inactivity needs to be recommended. The typical stability of mesh reconstructions of 50–150 N [30, 31] would allow a reconstruction size of 35–100 cm^2^ under consideration of the maximal physiologic intra-abdominal pressure of 14,000 N/m^2^; therefore, even without the stabilizing effect of peri-reconstructional soft tissue a properly executed mesh reconstruction is immediately stable and withstands pressure peaks due to coughing, pressing or heavy lifting.

Tolver et al. [19] counseled patients about a 1-day expected convalescence, leading to a resumption of work and ADL after 3–5 days without any negative consequences. Even this recommendation is, strictly speaking, debatable, but its consequent application would lead to an enormous reduction of socio-economic consequences of groin hernia.

Statements

**Table:** 

Level 1B	No specific period of physical inactivity is required after groin hernia repair.

**Recommendations**

**Table:** 

Grade B	The patient’s individual wish after counseling is to be respected and facilitated, e.g., by generous analgesics prescription; however, extended periods of sick-leave are usually not necessary and should not be supported

**In which way, if any, does convalescence pertain to the choice of surgical procedure?**

It is widely accepted and has been shown in numerous original articles and reviews that endoscopic hernia repair is associated with less postoperative pain and a reduced period of vocational and recreational downtime [18, 20, 32–49]. Due to the aforementioned substantial variation of actual periods of return to work and ADL, the benefit cannot be determined exactly; however, the differences are sufficiently pronounced and homogenous to warrant the recommendation of endoscopic techniques with regard to convalescence.

Statements

**Table:** 

Level 1A	Postoperative pain is less pronounced after endoscopic as compared to open hernia repair.
	Endoscopy hernia surgery is associated with shorter vocational downtime and earlier resumption of ADL as compared to open hernia repair.

**Recommendations**

**Table:** 

Grade B	With respect to convalescence, endoscopic hernia repair is preferable over open techniques.

**Comments**

All recommendations given in this chapter only apply to the conventional “heavy” (or small pore) mesh techniques since convalescence data for lightweight (or large-pore) mesh are not yet available. However, since there appear to be no differences in recurrence risk depending on mesh pore size [50] we provisionally assume that the recommendations are also applicable to large pore mesh techniques.

## References (in parentheses graduation of evidence)

1. Hendry PO, Paterson-Brown S, de Beaux A (2008) Work related aspects of inguinal hernia: a literature review. Surgeon 6:361–365. (**2A**)
2. Robertson GS, Burton PR, Haynes IG (1993) How long do patients convalescence after inguinal herniorrhaphy? Current principles and practice. Ann R Coll Surg Engl 75:30–33. (**4**)
3. Grewal P (2013) Survey of post-operative instructions after inguinal hernia repair in England in 2012. Hernia 18:269–272 (**4**)
4. Buhck H, Untied M, Bechstein WO (2012) Evidence-based assessment of the period of physical inactivity required after inguinal herniotomy. Langenbeck’s Arch Surg 397:1209–1214. (**2A**)
5. Simons MP, Aufenacker T, Bay-Nielsen M, Bouillot JL, Campanelli G, Conze J, de Lange D, Fortelny R, Heikkinen T, Kingsnorth A, Kukleta J, Morales-Conde S, Nordin P, Schumpelick V, Smedberg S, Smietanski M, Weber G, Miserez M (2009) European Hernia Society guidelines on the treatment of inguinal hernia in adult patients. Hernia 13:343–403. (**1A**)
6. McIntosh A, Hutchinson A, Roberts A, Withers H (2000) Evidence-based management of groin hernia in primary care—a systematic review. Fam Pract 17:442–447. (**2A**)
7. Bay-Nielsen M, Bisgaard T (2009) Rekonvalescens og sygemelding efter operation for lyskebrok. [Convalescence and sick leave following inguinal hernia repair]. Ugeskr Laeger 171:2899–2901. (**5**)
8. Lucht M (2008) Gesicherte Erkenntnisse zur Notwendigkeit einer körperlichen Schonung nach Leistenhernienoperation—Eine Evidenzbasierte Literaturstudie. Dissertation, Johann Wolfgang Goethe-Universität, Frankfurt/Main. (**2A**)
9. Kavic MS (2005) Hernia repair: 2005. A reflection. Hernia 9:308–309. (**5**)
10. McCormack K, Wake B, Perez J, Fraser C, Cook J, McIntosh E, Vale L, Grant A (2005) Laparoscopic surgery for inguinal hernia repair: systematic review of effectiveness and economic evaluation. Health Technol Assess 9:1–203, iii–iv. (**2A**)
11. Bourke JB, Taylor M (1978) The clinical and economic effects of early return to work after elective inguinal hernia repair. Br J Surg 65:728–731 (**1B**)
12. Bourke JB, Lear PA, Taylor M (1981) Effect of early return to work after elective repair of inguinal hernia: Clinical and financial consequences at one year and three years. Lancet 2:623–625. (**1B**)
13. Taylor EW, Dewar EP (1983) Early return to work after repair of a unilateral inguinal hernia. Br J Surg 70:599–600. (**1B**)
14. Amid PK, Lichtenstein IL (1998) Long-term result and current status of the Lichtenstein open tension-free hernioplasty. Hernia 2:89–94. (**4**)
15. Quilici PJ, Greaney EM, Jr, Quilici J, Anderson S (2000) Laparoscopic inguinal hernia repair: optimal technical variations and results in 1700 cases. Am Surg 66:848–852 (**4**)
16. Bay-Nielsen M, Thomsen H, Andersen FH, Bendix JH, Sørensen OK, Skovgaard N, Kehlet H (2004) Convalescence after inguinal herniorrhaphy. Br J Surg 91:362–367 (**2C**)
17. Bauer H, Fellmann E, Hermanek P, Hübner M, Jungnickel H, Kraas E, Ogasa J, Rückert K, Rümmelein D, Siefers H-F (2004) Leistenhernie. Bundesgeschäftsstelle für Qualitätssicherung (BQS); Düsseldorf. http://www.bqs-qualitaetsreport.de/2003/ergebnisse/leistungsbereiche/leistenhernie. Accessed 28 Dec 2012. (**2C**)
18. Lal P, Kajla RK, Chander J, Saha R, Ramteke VK (2003) Randomized controlled study of laparoscopic total extraperitoneal versus open Lichtenstein inguinal hernia repair. Surg Endosc 17:850–856. (**2B**)
19. Tolver MA, Strandfelt P, Forsberg G, Hjørne FP, Rosenberg J, Bisgaard T (2012) Determinants of a short convalescence after laparoscopic transabdominal preperitoneal inguinal hernia repair. Surgery 151:556–563. (**4**)
20. Callesen T (2003) Inguinal hernia repair: anesthesia, pain and convalescence. Dan Med Bull 50:203–218. (**5**)
21. Bay-Nielsen M, Thomsen H, Andersen FH, Bendix JH, Sorensen OK, Skovgaard N, Kehlet H (2004) Convalescence after inguinal herniorrhaphy. Br J Surg 91:362–367. (**2B**)
22. Ambach R, Weiss W, Sexton JL, Russo A (2000) Back to work more quickly after an inguinal hernia repair. Mil Med 165:747–750. (**4**)
23. Jones KR, Burney RE, Peterson M, Christy B (2001) Return to work after inguinal hernia repair. Surgery 129:128–135. (**4**)
24. Salcedo-Wasicek MC, Thirlby RC (1995) Postoperative course after inguinal herniorrhaphy. A case–controlled comparison of patients receiving workers’ compensation vs patients with commercial insurance. Arch Surg 130:29–32
25. McLauchlan GJ, Macintyre IM (1995) Return to work after laparoscopic cholecystectomy. Br J Surg 82:239–241. (**5**)
26. Bowley DM, Butler M, Shaw S, Kingsnorth AN (2003) Dispositional pessimism predicts delayed return to normal activities after inguinal hernia operation. Surgery 133:141–146. (**4**)
27. Tolver MA, Rosenberg J, Bisgaard T (2012) Early pain after laparoscopic inguinal hernia repair. A qualitative systematic review. Acta Anaesthesiol Scand 56:549–557. (**1A**)
28. Bay-Nielsen M, Perkins FM, Kehlet H (2001) Pain and functional impairment 1 year after inguinal herniorrhaphy: a nationwide questionnaire study. Ann Surg 233:1–7. (**2C**)
29. Kumar S, Wilson RG, Nixon SJ, Macintyre IM (2002) Chronic pain after laparoscopic and open mesh repair of groin hernia. Br J Surg 89:1476–1479. (**4**)
30. van’t Riet M, de Vos van Steenwijk PJ, Kleinrensink GJ, Steyerberg EW, Bonjer HJ (2002) Tensile strength of mesh fixation methods in laparoscopic incisional hernia repair. Surg Endosc 16:1713–1716. (**5**)
31. Hollinsky C, Göbl S (1999) Bursting strength evaluation after different types of mesh fixation in laparoscopic herniorrhaphy. Surg Endosc 13:958–961. (**5**)
32. Bittner R, Arregui ME, Bisgaard T, Dudai M, Ferzli GS, Fitzgibbons RJ, Fortelny RH, Klinge U, Kockerling F, Kuhry E, Kukleta J, Lomanto D, Misra MC, Montgomery A, Morales-Conde S, Reinpold W, Rosenberg J, Sauerland S, Schug-Pass C, Singh K, Timoney M, Weyhe D, Chowbey P (2011) Guidelines for laparoscopic (TAPP) and endoscopic (TEP) treatment of inguinal hernia [International Endohernia Society (IEHS)]. Surg Endosc 25:2773–2843. (**1A**)
33. Aasvang EK, Gmaehle E, Hansen JB, Gmaehle B, Forman JL, Schwarz J, Bittner R, Kehlet H (2010) Predictive risk factors for persistent postherniotomy pain. Anesthesiology 112:957–969. (**4**)
34. Schmedt CG, Sauerland S, Bittner R (2005) Comparison of endoscopic procedures vs Lichtenstein and other open mesh techniques for inguinal hernia repair: a meta-analysis of randomized controlled trials. Surg Endosc 19:188–199. (**1A**)
35. Dirksen CD, Beets GL, Go PM, Geisler FE, Baeten CG, Kootstra G (1998) Bassini repair compared with laparoscopic repair for primary inguinal hernia: a randomised controlled trial. Eur J Surg 164:439–447. (**2B**)
36. McCormack K, Scott NW, Go PM, Ross S, Grant AM (2003) Laparoscopic techniques versus open techniques for inguinal hernia repair. Cochrane Database Syst Rev CD001785. (**1A**)
37. Gholghesaei M, Langeveld HR, Veldkamp R, Bonjer HJ (2005) Costs and quality of life after endoscopic repair of inguinal hernia vs open tension-free repair: a review. Surg Endosc 19:816–821. (**2A**)
38. Johansson B, Hallerbäck B, Glise H, Anesten B, Smedberg S, Román J (1999) Laparoscopic mesh versus open preperitoneal mesh versus conventional technique for inguinal hernia repair: a randomized multicenter trial (SCUR Hernia Repair Study). Ann Surg 230:225–231. (**1B**)
39. Kuhry E, van Veen RN, Langeveld HR, Steyerberg EW, Jeekel J, Bonjer HJ (2007) Open or endoscopic total extraperitoneal inguinal hernia repair? A systematic review. Surg Endosc 21:161–166. (**1A**)
40. Mahon D, Decadt B, Rhodes M (2003) Prospective randomized trial of laparoscopic (transabdominal preperitoneal) vs open (mesh) repair for bilateral and recurrent inguinal hernia. Surg Endosc 17:1386–1390. (**2B**)
41. Memon MA, Cooper NJ, Memon B, Memon MI, Abrams KR (2003) Meta-analysis of randomized clinical trials comparing open and laparoscopic inguinal hernia repair. Br J Surg 90:1479–1492. (**2A**)
42. Neumayer L, Giobbie-Hurder A, Jonasson O, Fitzgibbons R, Jr., Dunlop D, Gibbs J, Reda D, Henderson W (2004) Open mesh versus laparoscopic mesh repair of inguinal hernia. N Engl J Med 350:1819–1827. (**1B**)
43. Reuben B, Neumayer L (2006) Surgical management of inguinal hernia. Adv Surg 40:299–317. (**5**)
44. Savarise MT, Simpson JP, Moore JM, Leis VM (2001) Improved functional outcome and more rapid return to normal activity following laparoscopic hernia repair. Surg Endosc 15:574–578. (**4**)
45. Schwab JR, Beaird DA, Ramshaw BJ, Franklin JS, Duncan TD, Wilson RA, Miller J, Mason EM (2002) After 10 years and 1903 inguinal hernias, what is the outcome for the laparoscopic repair? Surg Endosc 16:1201–1206. (**4**)
46. Stoker DL, Spiegelhalter DJ, Singh R, Wellwood JM (1994) Laparoscopic versus open inguinal hernia repair: randomised prospective trial. Lancet 343:1243–1245. (**1B**)
47. Tanović H, Mesihović R, Muhović S (2005) Randomized trial of TEP laparoscopic hernioplasty versus Bassni inguinal hernia repair. Med Arh 59:214–216. (**2B**)
48. Tanphiphat C, Tanprayoon T, Sangsubhan C, Chatamra K (1998) Laparoscopic vs open inguinal hernia repair. A randomized, controlled trial. Surg Endosc 12:846–851. (**2B**)
49. Wellwood J, Sculpher MJ, Stoker D, Nicholls GJ, Geddes C, Whitehead A, Singh R, Spiegelhalter D (1998) Randomised controlled trial of laparoscopic versus open mesh repair for inguinal hernia: outcome and cost. BMJ 317:103–110. (**2B**)
50. Sajid MS, Leaver C, Baig MK, Sains P (2012) Systematic review and meta-analysis of the use of lightweight versus heavyweight mesh in open inguinal hernia repair. Br J Surg 99:29–37. (**2A**)

## Chapter 16: Sportsman hernia—diagnosis and treatment

### Salvador Morales-Conde/Moshe Dudai, Reinhard Bittner

**Search terms**: Sportsmen HERNIA, Sport hernia, Athletes hernia, Athletes Pubalgia, Groin injury/treatment, Surgery, Technique, Repair, Surgical finding, Imaging, Pathology, Diagnosis, Etiology, Results, Complications

**Search machines**

PubMed; Medline.

**Publications**

Three new papers level 1 and 2 were identified.

**Diagnostic procedures**

One new, supplementary statement.

**Table:** 

Level 2B	CT scan has high accuracy in detecting posterior wall deficiency (PWD. (**new**)

No new recommendations.

**Indication for surgery**

New statements—identical to previous except statements below.

**Table:** 

Level 1B	Surgery (endoscopic placement of retropubic mesh) is more efficient than conservative therapy for the treatment of sportsman’s hernia. (**stronger evidence**).
	In Sportsman’s hernia the results of surgical repair to the posterior inguinal wall are excellent. (**stronger evidence**).
	For conservative treatment the use of radiofrequency denervation of both ilio-inguinal nerve and inguinal ligament in the treatment of refractory Sportsman’s Hernia is safe and efficacious at least in the short term, and is superior to anesthetic/steroid injection. (**new**).

New recommendations—identical to previous except recommendations below.

**Table:** 

Grade A	Endoscopic placement of retropubic mesh must be considered a serious option for Sportsman hernia. (**stronger evidence**).
	For conservative treatment of refractory Sportsman’s hernia, radiofrequency denervation of both ilio-inguinal nerve and inguinal ligament must be considered, in the short term, an alternative to anesthetic/steroid injection. (**new**).

**Comments**

One paper with level of evidence 2 has been published since 2009 based on the diagnostic procedures of sportsmen hernias [1]. Regarding treatment two level 1 studies are available: Comin [2] has published a study comparing radiofrequency denervation of both the ilio-inguinal nerve and inguinal ligament to desensitize the groin region and enable the athlete to become pain-free. This therapy was compared with local anesthetics (Bupivacaine) and steroid (Trimacinolone) injection, showing that the use of radiofrequency denervation is safe and efficacious at least in the short term, being superior to unaesthetic/steroid injection.

Regarding surgery, Paajanen et al. [3] compared conservative treatment to endoscopic mesh repair on 60 patients with a diagnosis of chronic groin pain and suspected sportsman’s hernia. Operative repair was more effective than non-operative treatment to decrease chronic groin pain after 1 month and up to 12 months of follow-up. Of the 30 athletes who underwent operation, 90 % returned to sports activities after 3 months of convalescence compared to 27 % of the 30 athletes in the non-operative group.

## References (in parentheses graduation of evidence)

1. Garvey JF (2012) Computed tomography scan diagnosis of occult hernia. Hernia 16:307–314. (**2B**)
2. Comin J, Obaid H, Lammers G, Moore J, Wotherspoon M, Connell D (2013) Radiofrequency denervation of the inguinal ligament for the treatment of ‘Sportsman’s Hernia’: a pilot study. Br J Sports Med 47:380–386. (**1B**)
3. Paajanen H, Brinck T, Hermunen H, Airo I (2011) Laparoscopic surgery for chronic groin pain in athletes is more effective than nonoperative treatment: a randomized clinical trial with magnetic resonance imaging of 60 patients with sportsman’s hernia (athletic pubalgia). Surgery 150:99–107. (**1B**)

## Chapter 17: Evidence based training for endoscopic/laparoscopic hernia repair (New chapter)

### Juliane Bingener

**Search terms**: Academic Medical Centers. *Clinical Competence. *Computer Simulation. *Computer-Assisted Instruction. *Curriculum. Education, Medical, Graduate/mt [Methods]. Education, Medical, Undergraduate/mt [Methods]. Female. Hernia, Inguinal/su [Surgery]. *Herniorrhaphy/ed [Education]. Herniorrhaphy/mt [Methods]. Humans. *Laparoscopy/ed [Education]. *Learning. Male. Medical Staff, Hospital/ed [Education]. Program Evaluation. Retroperitoneal Space/su [Surgery]. Time Factors. United States. Adult. Aged. Analysis of Variance. *Computer Simulation. *Computer-Assisted Instruction. Female. General Surgery. *Hernia, Inguinal/su [Surgery]. Hospitals, University. Humans. *Internship and Residency. *Laparoscopy. Length of Stay. Linear Models. Male. Middle Aged. Patient Satisfaction. Single-Blind Method. Time Factors. Treatment Outcome. User-Computer Interface.

**Search machines**

PubMed/Ovid MEDLINE/Ovid EMBASE/Web of Science/Scopus.

**Publications**

Following the above MESH terms, 46 abstracts resulted from the search and were reviewed. Of those, 24 full papers were reviewed. Seven papers were excluded as they only described mathematical models underlying virtual reality (VR) simulation for hernia repair. Five meta-analysis and systematic reviews, two randomized controlled trials, [10] prospective cohort studies were included.

**Introduction**

Laparoscopic inguinal hernia repair (LIHR) is an advanced laparoscopic procedure with a long learning curve, up to 250 procedures to proficiency [1, 2]. Zendejas et al. showed that simulation training leads to improved outcomes for patients undergoing laparoscopic inguinal hernia repair [3]. Simulation training tools and programs exist for both general laparoscopic task training and for procedure specific training. In the United States, surgeons now have to obtain a cognitive and general technical skills certification, the fundamentals of laparoscopic surgery (FLS), to be eligible for certification by the American Board of Surgery.

Beyond general task training, laparoscopic inguinal hernia specific trainers have been developed. Concepts exist on the low-tech box trainer platform, cadaveric tissue or the high tech virtual reality platform [4–7]. Low cost trainer boxes for laparoscopic inguinal hernia repair have been developed [4, 5]. They have face validity [5] and improve skills [4]. On review of the literature to date, no studies were encountered using computer simulated inguinal hernia repair for training. Along with the technical skills trainers, surgical educators have been interested in developing training curricula and assessment tools specific to inguinal hernia repair [8–10]. In addition, pathways to teach cognitive components and surgical decision making have been evaluated [11, 12].

After review of the above studies, we can make the following statements regarding levels of evidence and recommendations.

**Statements**

**Table:** 

Level 1A	Simulation training improves trainee satisfaction, trainee knowledge, time and process measure of skills, behaviors, compared to no training and to non-simulation training.
Level 1A	Computer simulation and box trainers improve operative performance.
	Box training is as effective as computer simulation and results in higher learner satisfaction
Level 1B	Cognitive training plus mastery learning on box trainers improves patient outcome
Level 2B	GOALS-GH is an objective and valid measure of skills required to perform LIHR (TAPP and TEP).
	Training on fresh frozen cadaver has higher face validity than training on a VR trainer.

**Recommendations**

**Table:** 

Grade A	A simulation trainer should be available to all learners to improve operative performance.
	At the current time, box trainers are preferred over computer-assisted simulation for inguinal hernia repair.
Grade B	A proficiency-based curriculum for the available trainer tool should be established to improve patient outcomes.
	A validated assessment tool should be used to assess proficiency.

**Comments**

A recent study linked surgical skill to patient outcome after bariatric surgery for surgeons in practice, underlining the increased focus on technical proficiency even beyond the training phase. Here we reviewed the literature to provide recommendations how to set up deliberate practice opportunities for trainees to become experts [13]. It is clear that beyond the presence of a training tool, a cognitive and technical training curriculum is vital to improve surgeon skills and patient outcomes.

Faculty involvement does not have to be extensive, as research on feedback in other surgical areas suggests [14–16]. Faculty feedback is moderately effective for learner skills training. Terminal feedback is more effective than concurrent feedback for learners’ skills retention (level 2A evidence). A small prospective study reported that providing video-based cognitive and technical instruction along with training parameters and a feedback session after a 6-week period increased practice frequency and improved skills [17].

## References (in parentheses graduation of evidence)

1. Zendejas B, Onkendi EO, Brahmbhatt RD, Lohse CM, Greenlee SM, Farley DR (2011) Long-term outcomes of laparoscopic totally extraperitoneal inguinal hernia repairs performed by supervised surgical trainees. Am J Surg 201:379–383; discussion 383–374. (**2C**)
2. Neumayer L, Giobbie-Hurder A, Jonasson O, Fitzgibbons R, Jr, Dunlop D, Gibbs J, Reda D, Henderson W (2004) Open mesh versus laparoscopic mesh repair of inguinal hernia. N Engl J Med 350:1819–1827. (**1B**)
3. Zendejas B, Cook DA, Bingener J, Huebner M, Dunn WF, Sarr MG, Farley DR (2011) Simulation-based mastery learning improves patient outcomes in laparoscopic inguinal hernia repair: a randomized controlled trial. Ann Surg 254:502–509; discussion 509–511. (**1B**)
4. Jain M, Tantia O, Khanna S, Sen B, Sasmal PK (2009) Hernia endotrainer: results of training on self-designed hernia trainer box. J Laparoendosc Adv Surg Tech A 19:535–540. (**2B**)
5. Kurashima Y, Feldman L, Al-Sabah S, Kaneva P, Fried G, Vassiliou M (2011) A novel low-cost simulator for laparoscopic inguinal hernia repair. Surg Innov 18:171–175. (**2B**)
6. Devarajan V, Wang X, Shen Y, Eberhart R, Watson MJ, Jones D, Villegas L (2006) A novel laparoscopic mesh placement part task trainer. Int J Med Robot 2:312–320. (**5**)
7. Devarajan V, Scott D, Jones D, Rege R, Eberhart R, Lindahl C, Tanguy P, Fernandez R (2001) Bimanual haptic workstation for laparoscopic surgery simulation. Studies in health technology and informatics 81:126–128. (**5**)
8. Kurashima Y, Feldman LS, Al-Sabah S, Kaneva PA, Fried GM, Vassiliou MC (2011) A tool for training and evaluation of laparoscopic inguinal hernia repair: the Global Operative Assessment Of Laparoscopic Skills-Groin Hernia (GOALS-GH). Am J Surg 201:54–61. (**2B**)
9. Adrales GL, Park AE, Chu UB, Witzke DB, Donnelly MB, Hoskins JD, Mastrangelo MJ, Jr, Gandsas A (2003) A valid method of laparoscopic simulation training and competence assessment. J Surg Res 114:156–162. (**2C**)
10. Adrales GL, Chu UB, Witzke DB, Donnelly MB, Hoskins D, Mastrangelo MJ, Jr., Gandsas A, Park AE (2003) Evaluating minimally invasive surgery training using low-cost mechanical simulations. Surg Endosc 17:580–585. (**2C**)
11. Pugh C, Plachta S, Auyang E, Pryor A, Hungness E (2010) Outcome measures for surgical simulators: is the focus on technical skills the best approach? Surgery 147:646–654. (**2C**)
12. Pugh CM, DaRosa DA, Santacaterina S, Clark RE (2011) Faculty evaluation of simulation-based modules for assessment of intraoperative decision making. Surgery 149:534–542. (**2B**)
13. Ericsson K (1993) The role of deliberate practice in the acquisition of expert performance. Psychol Rev 100:363–406.(**5**)
14. Brydges R, Nair P, Ma I, Shanks D, Hatala R (2012) Directed self-regulated learning versus instructor-regulated learning in simulation training. Med Educ 46:648–656. (**1B**)
15. Walsh CM, Ling SC, Wang CS, Carnahan H (2009) Concurrent versus terminal feedback: it may be better to wait. Acad Med 84:S54–57. (**2B**)
16. Jensen AR, Wright AS, Levy AE, McIntyre LK, Foy HM, Pellegrini CA, Horvath KD, Anastakis DJ (2009) Acquiring basic surgical skills: is a faculty mentor really needed? Am J Surg 197:82–88. (**1B**)
17. Ruparel R, Zendejas B, Onkendi EO, Al Jamal YN, Farley DR, Bingener J (2013) Mentor-guided self directed learning curriculum improves practice habits among 2nd year surgical residents (abstract, program booklet) American College of Surgeons Annual Clinical Congress, October 6–10, 2013, Washington, DC. (**2B**)

## Chapter 18: Costs in endoscopic/laparoscopic and open hernia surgery (New chapter)

### Reinhard Bittner, Ferdinand Köckerling

**Search terms**: “costs” and “inguinal hernia repair”, “costs” and “laparoscopic inguinal hernia repair”, “cost-effectiveness” and “laparoscopic inguinal hernia repair”, “cost benefit” and “laparoscopic inguinal hernia repair”, “quality of life” and “laparoscopic inguinal hernia repair”, “value for money” and “hernia surgery”, “ QALY” and “hernia surgery”.

**Search machines**

Pubmed, Medline.

**Number of publications**

A total of 333 papers were identified. Due to the reason that the guidelines should focus on the comparison “open flat mesh vs. laparoscopic mesh repair 223 had to be to exclude because of not relevant to this topic, double publication, or referring to pediatric hernia surgery. After reading the abstracts of the remaining 110 papers again 43 papers were excluded because of not reporting any cost calculations. After reading the full text of the 67 papers left, 45 papers were found useful for the development of the presented guidelines.

**Questions**

- Does hernia surgery offer value for money, is there a difference between open and laparoscopic surgery?
- Which factors are influencing the costs in inguinal hernia surgery?
- Which of the cost factors the surgeon is able to influence?
- Are there possibilities to reduce the costs?
- Are there differences in direct costs (hospital) between open and laparoscopic repair?
- Are there differences in indirect costs (societal) between open and laparoscopic repair?
- Are there differences in the costs per QALY between open and laparoscopic surgery?
- Which measures can be recommended for cost reduction?
- Can additional measures be recommended for practitioners who work in countries with limited health care resources?

Statements

**Table:** 

Level 1A	When using disposable trocars and instruments direct costs (hospital) are higher for laparoscopic inguinal hernia repair.
	Total costs (hospital and societal) are lower for laparoscopic inguinal hernia repair compared to open.
	Operation time is a cost-relevant factor.
	Time for anesthesia is a cost-relevant factor.
	Experience and quality of performance are cost-relevant factors.
	Simulator-training may improve quality of performance.
Level 2C	Hernia surgery is cost-effective. It may be superior to “watchful waiting” in the long run.
	Laparoscopic hernia surgery offers a higher cost-utility compared to open.
	Hospitals costs for laparoscopic hernia repair may be similar or lower compared to open but there is a large variation in cost per QALY generated by individual providers.
	In hospitals with a high case load costs are lower.

**Recommendations**

**Table:** 

Grade A	Non-disposable trocars and instruments must be considered.
	Non-fixation techniques should be considered. Use of no or indigenous balloon must be considered.
	Operative performance and education of the surgeons must be improved.
	To shorten the learning curve of traineesurgeons, simulator training should be introduced.
Grade B	In hernia disease surgery might be superior to “watchful waiting”.
	From the point of cost-utility laparoscopic inguinal hernia repair may be considered.
	To enhance the case load centralization of hernia surgery should be considered.

**Comments**

Cost calculations in treatment of inguinal hernias are difficult to perform mainly due to the multitude of factors having some influence on the costs. In 2006 a large randomized controlled study (RCT) showed that at 2 years “watchful waiting” (WW) is a cost-effective treatment option for men with minimal or no groin hernia symptoms [1]. But 7 years later the same group of authors found a long-term crossover rate of 68 % and concluded that although WW is a reasonable and safe strategy, symptoms will likely progress and an operation will be needed eventually [2]. In accordance with this long-term result a large register study from UK recently published demonstrated that hernia surgery offers value for money [3]. Moreover these authors found laparoscopic repair more cost-effective and providing less money per quality adjusted live years (QALY) in comparison to open surgery. Two previously published comprehensive reviews reported similar results [4, 5].

With regard to hospital costs only nearly all RCT’s show higher costs for the laparoscopic repair (TAPP, TEP) [6–32]. But the reliability of some of these studies should be scrutinized. Long operating times (>60 min) [7, 8, 10, 14, 15, 19, 24, 31], high recurrence rates for laparoscopic repair (10 %) [33] and high conversion rate (6–10 %) [21, 27, 29] reported indicate lack of experience. Moreover studies not mentioning the kind of instruments and materials are useless for cost calculations. In contrast to these RCT’s when analyzing routine administrative highly standardized, patient-level cost data (collected in 15 German hospitals participating in the national cost data study) Wittenbecher et al. 2013 [34] found lower costs for TEP/TAPP and concluded that laparoscopic approaches are not necessarily associated with higher hospital resource consumption than open mesh repair.

These conflicting data demonstrate clearly that cost calculations in hernia surgery are complex because of the nearly countless number of cost-relevant variables. These factors may be dependent on the patient, the pathology of the hernia, type of anesthesia, case load of hernias per year, type of procedure, skills of the surgeon, operating time, materials, meshes, type of fixation or no fixation, complications, setting in which operation is performed (ambulatory, size of hospital/institution, country, region), number of postoperative visits/home care, time of sick leave, outcome (recurrence rate, quality of life), salaries of the personnel, depreciation of equipment, and an appropriate share of the costs of the most relevant support departments: administration, house keeping, cleaning, sterilization, equipment maintenance. According to that apparently countless number of factors the published data with regard to costs show a huge range from about 126 US-$ to more than 4116 US $ [3, 20]. Moreover even within one institution there is a large variation in costs generated by individual providers [3]. Only a few of these factors may be influenced by the surgeon. Operating time, quality of the surgical intervention as well as the choice of instruments and materials are directly under the responsibility of the surgeon [29, 30, 34, 35]. In most of the papers it is stated that the higher costs found in laparoscopic surgery is mainly a reflection of the greater use of expensive disposable equipment and longer operating time for laparoscopic hernia repair [5, 10, 12, 13, 15, 17, 20, 24, 27, 30]. Multiple sensitivity analyses demonstrated that when use of disposable trocars, graspers, preperitoneal balloon, and stapling devices (“tacker”) were included, direct costs and charges were significantly higher for laparoscopic hernia repair. On the other hand, in a large volume laparoscopic surgery center with minimal use of disposable instruments and avoidance of preperitoneal balloon and tackers for mesh fixation, the actual direct costs of laparoscopic repair are comparable to open repairs [24]. Controversially discussed are the use of low-cost meshes [36] and the use of indigenous dilatation balloons [37] for further cost reduction. But without doubt experience is a significant factor for decreasing operating time as well as the rate of complications, recurrences and long-term complaints like chronic pain [29, 30, 34, 38]. In so far surgical performance is directly correlated to quality of life and QALY’S.

Different to the results of the calculations of hospital costs (direct) nearly all RCT’s, systematic reviews, and meta-analysis prove that the societal costs(indirect) are less after laparoscopic repair mainly due to more rapid recovery and a shorter time of sick leave [4, 5, 7, 10–13, 15, 16, 19, 30, 35] when compared to open surgery.

In summary, up to now due to the higher hospital costs worldwide acceptance of laparoscopic hernia repair is low despite less pain and more rapid recovery in comparison to open surgery. Therefore cost containment measures are to consider like increase of the case load (more rapid depreciation of equipment costs, large experience) [39], shortening of the learning curve and improvement of surgical performance by standardizing the technique and systematic training [38, 40]. Other recommendations are using non-disposable trocars and instruments [24, 25, 41, 42, 43], avoidance of “tacker” fixation [44] and implantation of low-cost meshes [36, 45].

## References (in parentheses graduation of evidence)

1. Stroupe KT, Manheim LM, Luo P, Giobbie-Hurder A, Hynes DM, Jonasson O, Reda DJ, Gibbs JO, Dunlop DD, Fitzgibbons RJ Jr (2006) Tension-free repair versus watchful waiting for men with asymptomatic or minimally symptomatic inguinal hernias: a cost-effectiveness analysis. J Am Coll Surg 203(4):458–468. (**1B**)
2. Fitzgibbons RJ Jr, Ramanan B, Arya S, Turner SA, Li X, Gibbs JO, Reda DJ, Investigators of the Original Trial (2013) Long-term results of a randomized controlled trial of a nonoperative strategy (watchful waiting) for men with minimally symptomatic inguinal hernias. Ann Surg 258(3):508–515. (**1B**)
3. Coronini-Cronberg S, Appleby J, Thompson J (2013) Application of patient-reported outcome measures (PROMs) data to estimate cost-effectiveness of hernia surgery in England. J R Soc Med 106: 278–287. (**2C**)
4. Stylopoulos N, Gazelle GS, Rattner DW (2002) A cost-utility analysis of treatment options for inguinal hernia in 1,513,008 adult patients. Surg Endosc 17(2):180–189. (**1A**)
5. Gholghesaei M, Langeveld HR, Veldkamp R, Bonjer HJ (2005) Costs and quality of life after endoscopic repair of inguinal hernia vs open tension-free repair: a review. Surg Endosc 19(6):816–821. (**1A**)
6. Payne JH Jr, Grininger LM, Izawa MT, Podoll EF, Lindahl PJ, Balfour J (1994) Laparoscopic or open inguinal herniorrhaphy? A randomized prospective trial. Arch Surg 129(9):973–979; (**1B**)
7. Brooks DC (1994) A prospective comparison of laparoscopic and tension-free open herniorrhaphy. Arch Surg 129(4):361–366. (**2B**)
8. Lawrence K, McWhinnie D, Goodwin A, Gray A, Gordon J, Storie J, Britton J, Collin J (1995) Randomised controlled trial of laparoscopic versus open repair of inguinal hernia: early results. BMJ 311(7011):981–985. (**1B**)
9. Lawrence K, McWhinnie D, Goodwin A, Gray A, Gordon J, Storie J, Britton J, Collin J (1996) An economic evaluation of laparoscopic versus open inguinal hernia repair. J Public Health Med 18(1):41–48. (**1B**)
10. Liem MS, Halsema JA, van der Graaf Y, Schrijvers AJ, van Vroonhoven TJ (1997) Cost-effectiveness of extraperitoneal laparoscopic inguinal hernia repair: a randomized comparison with conventional herniorrhaphy. Coala trial group. Ann Surg 226(6):668–675(**1B**)
11. Kald A, Anderberg B, Carlsson P, Park PO, Smedh K (1997) Surgical outcome and cost-minimization-analyses of laparoscopic and open hernia repair: a randomised prospective trial with one year follow up. Eur J Surg 163(7):505–510. (**1B**)
12. Heikkinen TJ, Haukipuro K, Hulkko A (1998) A cost and outcome comparison between laparoscopic and Lichtenstein hernia operations in a day-case unit. A randomized prospective study. Surg Endosc 12 (10):1199–1203. (**1B**)
13. Wellwood J, Sculpher MJ, Stoker D, Nicholls GJ, Geddes C, Whitehead A, Singh R, Spiegelhalter D (1998) Randomised controlled trial of laparoscopic versus open mesh repair for inguinal hernia: outcome and cost. BMJ 317(7151):103–110. (**1B**)
14. Paganini AM, Lezoche E, Carle F, Carlei F, Favretti F, Feliciotti F, Gesuita R, Guerrieri M, Lomanto D, Nardovino M, Panti M, Ribichini P, Sarli L, Sottili M, Tamburini A, Taschieri A (1998) A randomized, controlled, clinical study of laparoscopic vs open tension-free inguinal hernia repair. Surg Endosc 12(7):979–986. (**1B**)
15. Johansson B, Hallerbäck B, Glise H, Anesten B, Smedberg S, Román J (1999) Laparoscopic mesh versus open preperitoneal mesh versus conventional technique for inguinal hernia repair: a randomized multicenter trial (SCUR Hernia Repair Study. Ann Surg 230(2):225–231. (**1B**)
16. Jönsson B, Zethraeus N (2000) Costs and benefits of laparoscopic surgery—a review of the literature. Eur J Surg Suppl(585):48–56. (**1A**)
17. Medical Research Council Laparoscopic Groin Hernia Trial Group (2001) Cost-utility analysis of open versus laparoscopic groinhernia repair: results from a multicentre randomized clinical trial. Br J Surg 88(5):653–61. (**1B**)
18. Papachristou EA, Mitselou MF, Finokaliotis ND (2002) Surgical outcome and hospital cost analyses of laparoscopic and open tension-free hernia repair. Hernia. 6(2):68–72. (**3**)
19. Schneider BE, Castillo JM, Villegas L, Scott DJ, Jones DB (2003) Laparoscopic totally extraperitoneal versus Lichtenstein herniorrhaphy: cost comparison at teaching hospitals. Surg Laparosc Endosc Percutan Tech 13(4):261–267. (**3**)
20. Vale L, Ludbrook A, Grant A (2003) Assessing the costs and consequences of laparoscopic vs. open methods of groin hernia repair: a systematic review. Surg Endosc 17(6):844–849. (**1A**)
21. Hildebrandt J, Levantin O (2003) Tension-free methods of surgery of primary inguinal hernias. Comparison of endoscopic, total extraperitoneal hernioplasty with the Lichtenstein operation. Chirurg 74(10):915–921. (**1B**)
22. Hahn S, Whitehead A (2003) An illustration of the modelling of cost and efficacy data from a clinical trial. Stat Med 22(6):1009–1024. (**1B**)
23. Anadol ZA, Ersoy E, Taneri F, Tekin E (2004) Outcome and cost comparison of laparoscopic transabdominal preperitoneal hernia repair versus Open Lichtenstein technique. J Laparoendosc Adv Surg Tech A 14(3):159–163. (**3**)
24. Khajanchee YS, Kenyon TA, Hansen PD, Swanström LL(2004) Economic evaluation of laparoscopic and open inguinal herniorrhaphies: the effect of cost-containment measures and internal hospital policy decisions on costs and charges. Hernia 8(3):196–202. (**2b**)
25. McCormack K, Wake B, Perez J, Fraser C, Cook J, McIntosh E, Vale L, Grant A (2005) Laparoscopic surgery for inguinal hernia repair: systematic review of effectiveness and economic evaluation. Health Technol Assess 9(14):1–203. (**1A**)
26. Hynes DM, Stroupe KT, Luo P, Giobbie-Hurder A, Reda D, Kraft M, Itani K, Fitzgibbons R, Jonasson O, Neumayer L (2006) Cost effectiveness of laparoscopic versus open mesh hernia operation: results of a Department of Veterans Affairs randomized clinical trial. J Am Coll Surg 203(4):447–457. (**1B**)
27. Butler RE, Burke R, Schneider JJ, Brar H, Lucha PA Jr (2007) The economic impact of laparoscopic inguinal hernia repair: results of a double-blinded, prospective, randomized trial. Surg Endosc 21(3):387–390. (**1B**)
28. Kuhry E, van Veen RN, Langeveld HR, Steyerberg EW, Jeekel J, Bonjer HJ (2007) Open or endoscopic total extraperitoneal inguinal hernia repair? A systematic review. Surg Endosc 21(2):161–166. (**1A**)
29. Langeveld HR, van’t Riet M, Weidema WF, Stassen LP, Steyerberg EW, Lange J, Bonjer HJ, Jeekel J (2010) Total extraperitoneal inguinal hernia repair compared with Lichtenstein (the LEVEL-Trial): a randomized controlled trial. Ann Surg 251(5):819–824. (**1B**)
30. Eklund A, Carlsson P, Rosenblad A, Montgomery A, Bergkvist L, Rudberg C (2010) Swedish Multicentre Trial of Inguinal Hernia Repair by Laparoscopy (SMIL) study group. Br J Surg. 97(5):765–771. (**1B**)
31. Smart P, Castles L (2012) Quantifying the costs of laparoscopic inguinal hernia repair. ANZ J Surg 82(11):809–812. (**3**)
32. Wang WJ, Chen JZ, Fang Q, Li JF, Jin PF, Li ZT (2013) Comparison of the effects of laparoscopic hernia repair and Lichtenstein tension-free hernia repair. J Laparoendosc Adv Surg Tech A 23(4):301–305. (**1B**)
33. Neumayer L, Giobbie-Hurder A, Jonasson O, Fitzgibbons R Jr, Dunlop D, Gibbs J, Reda D, Henderson W; Veterans Affairs Cooperative Studies Program 456 Investigators (2004) Open mesh versus laparoscopic mesh repair of inguinal hernia. N Engl J Med 350(18):1819–1827. (**1B**)
34. Wittenbecher F, Scheller-Kreinsen D, Röttger J, Busse R (2013) Comparison of hospital costs and length of stay associated with open-mesh, totally extraperitoneal inguinal hernia repair, and transabdominal preperitoneal inguinal hernia repair: an analysis of observational data using propensity score matching. Surg Endosc 27(4):1326–1333. (**2C**)
35. Aly O, Green A, Joy M, Wong CH, Al-Kandari A, Cheng S, Malik M (2011) Is laparoscopic inguinal hernia repair more effective than open repair? J Coll Physicians Surg Pak 21(5):291–296. (**1A**)
36. Yang J, Papandria D, Rhee D, Perry H, Abdullah F (2011) Low-cost mesh for inguinal hernia repair in resource-limited settings. Hernia 15(5):485–489. (**1A**)
37. Misra MC, Kumar S, Bansal VK (2008) Total extraperitoneal (TEP) mesh repair of inguinal hernia in the developing world: comparison of low-cost indigenous balloon dissection versus direct telescopic dissection: a prospective randomized controlled study. Surg Endosc 22(9):1947–1958. (**1B**)
38. Koperna T (2004) How long do we need teaching in the operating room? The true costs of achieving surgical routine. Langenbecks Arch Surg 389(3):204–208. (**3**)
39. Chatterjee S, Laxminarayan R (2013) Costs of surgical procedures in Indian hospitals. BMJ Open 20:3(6). pii: e002844.(**2C**)
40. Kurashima Y, Feldman LS, Kaneva PA, Fried GM, Bergman S, Demyttenaere SV, Li C, Vassiliou MC (2014) Simulation-based training improves the operative performance of totally extraperitoneal (TEP) laparoscopic inguinal hernia repair: a prospective randomized controlled trial. Surg Endosc 28(3):783–788. (**1B**)
41. Lau H, Lee F, Patil NG, Yuen WK (2002) Two hundred endoscopic extraperitoneal inguinal hernioplasties: cost containment by reusable instruments. Chin Med J (Engl) 115(6):888–891. (**3**)
42. Farinas LP, Griffen FD (2000) Cost containment and totally extraperitoneal laparoscopic herniorrhaphy. Surg Endosc 14(1):37–40. (**3**)
43. Basu S, Chandran S, Somers SS, Toh SK (2005) Cost-effective laparoscopic TEP inguinal hernia repair: the Portsmouth technique. Hernia 9(4):363–367. (**3**)
44. Taylor C, Layani L, Liew V, Ghusn M, Crampton N, White S (2008) Laparoscopic inguinal hernia repair without mesh fixation, early results of a large randomised clinical trial. Surg Endosc 22(3):757–762. (**1B**)
45. Cavallo JA, Ousley J, Barrett CD, Baalman S, Ward K, Borchardt M, Thomas JR, Perotti G, Frisella MM, Matthews BD (2014) A material cost-minimization analysis for hernia repairs and minor procedures during a surgical mission in the Dominican Republic. Surg Endosc 28(3):747–766. (**3**)

